# Supplementary material for: Predictors of cervical cancer screening uptake in two districts of Central Uganda
Source: PLoS One. 2020 Dec 3;15(12):e0243281. doi: 10.1371/journal.pone.0243281 (PMC7714132; doi:10.1371/journal.pone.0243281)
Supplement: S1 File — (DOCX) [file pone.0243281.s002.docx]

**Esonga eziretera abakyala okwetaba mu kuziyiza kokolo womumwa gwa nabaana mu masekate ga Uganda**

**Ekiwandiiko ekiraaga okukiriza okwetaba mu kunonyereza eri abakyala abali wakati w’emyaka 25-49 mu Nakasongola ne Wakiso district.**

**Okweyajula;-** Nkulamusiza ssebo/nyabo, amanya nze _______________________________________ era nkola ne Isabirye Alone omuyizi ku ddala elya PhD okuva ku ssetendekero lya Makerere University. Mukaseera kano, tuli mukunonyereza ku bulwadde bwa kkokolo w’omumwa gwa nabaana nga essira mu kunonyereza tulitadde ku biteberezebwa okuziyiiza obulwadde bwa kokolo wa n’abaana mu bakyala abali wakati w’emyaka 25-49 awamu ne bawala babwe abali wakati w’emyaka 10-17 be babeera nabo.

**Ebitono-tono ku kunonyereza kuno**;- Kokolo w’omumwa gwa nabaana akosa nyo era wabulabe eri abakyala n’ebyobulamu mu Uganda. Ebivune ebyekwasa ku kokolo w’omumwa gwa nabaana biyiiza okukedezebwa singa ensonga eziretera abakyala okwetaba mu kubuziyiza zimanyibwa. Enddowoza n’okuwabulwa kwo kujjakubera kwamugaso mu kwongera okwetanira okumanya ku birina okukolebwa okuziyiiza kokolo w’omumwa gwa nabaana.

**Omulimo gwa eyettaba mu kunonyereza;-** Tujja ku buza abakyala 810 abali mu myaka 25-49 mu Wakiso ne Nakasongola districts. Oli omu kwabo abakyala abalondedwa okwettaba mukunonyereza kuno. Ojja kubuzibwa ebibuuzo ebyekwasaganyiza ku kokolo w’omumwa gwa nabaana wamu n’okuziyiza okutukwata. okunonyereza kuno kujja kutwala eddakika ana (40) ku atano (50).

**Obulabe/ n’ebirungi ebiri mu kwettaba mu kunonyereza kuno;-** Ebibuuzo ebimu bikwasa ensonyi. Bwemba nga kubuziza ekibuuzo kyona kyowulira nti tewaliyagadde oba toyagala kuddamu, ntegezaako jjakuba ngedda kwekyo ekikiddako. Nekirara, okunonyereza kuno kuyiiza obutakuyamba gwe nga omuntu, naye ebinaavamu bijja kuyamba okwongera okutereza mu mutindo gw’empereza eyobujjajabi obwekulusanyiza ku kwetangira kokolo w’omumwa gwa nabaana mu kitundu kino awamu n’ebifo ebirara.

**Obukumi ku byogenda okutuganba**;- Okuddamukwo kwona kwonotuwa kujjakuterekebwa ng’ekyama. Mu kufuba okukuuma ebyama bino, erinya lyo terijja kuwandiikibwa ku lupapula olunabuuzibwako, wabuula tujja kukozesa e namba eyenjawulo. Tujja kutereka ebiwandiiko bino mu kifo tubikuume butiribiri. Abakoozi abanakiriizibwa, b’ebajja okutunura mu biwandiiko bino kulw’okutegekka okumanyisibwa. Okumanyisibwa kuno kujja kukozesebwa kunonyereza kuno kwoka. Okumanyisibwa kujja kwanjulibwa mu bunji bwabwo si bwa muntu kinomu, mu binaava mu musomo, ne mu binatekebwa mulujjudde. Nga omusomo gwa PhD guwedde, ebibuuzo bijja kusanyizibwawo

**Edembe lyo;-** Osobola okusalawo okwettaba mu kunonyereza kuno oba neda. Kiri gwe okusalawo. Bwoba asozzewo obuttetaba mu musomo guno, tojja kuvunanibwa olwekyo. Naye, e ssubi lyaffe liri nti okiriiza okuddamu ebibuuzo kubanga enddowoza zo z’amugaso. Osobola okuyimiriza okwogerezeganya kuno e ssawa yona.

**Okufulumya ebinava mukunonyereza;-** Ebinaava mu kunonyereza kuno bijja kwajulibwa ku emu ku mikuutu gya radiyo eri mu kitundu kino.

**Olukusa okukola okunonyereza kuno;-** Omusomo guno gwakirizibwa ekitongole kya Makerere University ekivunanyizibwa kukunonyereza kumbera zaabantu (School of Social Sciences Research and ethics committee (MAHSS REC)). Olukuusa okukungaanya endowooza zamwe lwawebwa okuva mu kakiiko akatwala ebyekikugu ne tekinologiya mu Uganda (National Council for Science and Technology) wamu ne office y’obwa Presidenti. Olukuusa okukunganya endowooza zamwe lwafunibwa okuva mu office yo mubaka wa presidenti ku district (Resident District Commissioner (RDC)). Era, okunonyereza kwekumu kwakilizibwa abakulembeze abawansi mu kitundu kino.

**Amassimu n’ebibuuzo;-** Bw’oba olina ebibuuzo kati, nkusaba ombuuze. Bwo’oba olina ebibuuzo mu maaso eyo, ebikwatagana n’omusomo guno, Osobola okutukirira; **Isabirye Alone**; Department of Population Studies, School of Statistics and Planning, College of Business and management Sciences, Makerere University. esimu: 0774497873. Ng’oyagala okwogera n’omuntu yena okujjako abanonyerezi ku; bintu ebikwatagana n’okunonyereza kuno, edembe lyo, obulabe oba ensonga yoona eyinza okukukosa, bambi tukiriira: **Dr. Stella Neema** ,akulira- Makerere School of Social Sciences, Research Ethics Committee esimu: +256- 772 457576, E-mail: [sheisim@yahoo.com](mailto:sheisim@yahoo.com) Oba **Ssabawandiisi wa** Uganda National Council of Science and Technology, olugudo- Kimera. Ntinda akabox 6884 Kampala, Uganda, esimu: (256) 414 705500 Fax: +256-414-234579, Email: [info@uncst.go.ug](mailto:info@uncst.go.ug).

| Okiriiza okwetaba mu kunonyereza kuno? | YEE  W’ebazza omuntu oyo weyongereyo ku kitundu ekiddako…..1 | | | |
| --- | --- | --- | --- | --- |
|  | Neda  W’ebazza omuntu oyo okomekereeze embozzi…………...0 | | | |
| Abuzibwa akiriiza okubuuzibwa. | | 1 | Abuzibwa tt’akiriiza kubuuzibwa –Komekereeza embozi |  |

**EBIBUZO BYA ABAKYALA ABALINA EMYAKA 25-49 E NAKASONGOLA NE WAKISO DISTRICTS**

**Namba eyenjawulo yooyo abuzibwa________________________________**

| Amanya g’omusoyasoya:……………………………….  Signature/ omukono gwe……………………………………………..Olunaku…………………. |
| --- |

| **E SSAWA EZITANDIIKA** | **SSAWA** |  |  | **DAKIKA** |  |  |
| --- | --- | --- | --- | --- | --- | --- |

**Amateeka agafuga omubuuzi:**

| ***Teka*** |  | ***mu ka boxi ak’ekiddibwamu ky’olonze.*** |
| --- | --- | --- |

T’osoma biddibwamu okujjako nga endagiriro bwetyo bwesoma.

**Okumanya kubikukwatako**

| **NO.** | **EBIBUUZO N’EBISENGEJJA** | **EKYOKULODAKO** | **BUUKA** |
| --- | --- | --- | --- |
| 100A | Obera mukyalo oba mukibuga? | - Kyalo------------0 - Kibuga------------1 |  |
| 100B | District | - Wakiso------------1 - Nakasongola------2 |  |
| 100C | Division/ Egombolola |  |  |
| 100D | Ekyalo/ LC/ Ward |  |  |

**EKITUNDU 100: EBIKUKWATAKO**

| **No.** | **EBIBUUZO N’EBISENGEJJA** | **EKYOKULODAKO** | **BUUKA** |
| --- | --- | --- | --- |
| 101 | W’azalibwa mu mwezi n’omwaka ki? | ______/_____  Omwezi/ Omwaka |  |
| 101a | Walina emyaka emeka ku m’azalibwa go aggasebyeyo? | Emyaka |  |
| 102 | Wali ogenzeko ku ssomero?  **Bwekiba nti Yee**  Ddaala ki elyemisomo elisukulumu lye wa maliriza? | - Sasoma nakamu..…….1 - Primary………………..2 - O level…………………3 - Higher………………….4 - University……………....5 - Koosi…………………6 |  |
| 103 | Oli wa ddini ki? | - Mukatuliki…….…….…….1 - Muprotestant ........................2 - Musilamu ..………….....… 3 - Pentecostal……….…..... 4 - SDA……………...……. 5 - Ekirara (kiki).…...……..6 |  |
| 104 | Oli wa gwanga ki? | - Muganda . . . . …………..1 - Munyankole……………. 2 - Musoga . .. ……...….... . . 3 - Mukiga ….…………… . . 4 - Muruli . . ……………..…. 5 - Ekirara (kiki) …………...6 |  |

| 104a | Wali ofumbidwako oba n’obeera n’omusajja ng’abafumbo? | | - Yee……………..1 - Neda……………0 | |  |
| --- | --- | --- | --- | --- | --- |
| 104b | Oli namwandu, wanoba, oba wayawukana ne bawo/ mwagalwa wo? | | - Mufumbo…………………….1 - Namwandu …………………...2 - Nanoba……………..………….3 - Twayawukana………….......…..4 | |  |
| 105 | | Akulira amaaka gano /enyumba/enju/ eno wa kitonde ki? | | - Musajja………….1 - Mukazzi………..2 |  |
| 106 | | Mu bisera bino, wettaba mu mirimu egisasurwa? | | - Yee …………..1 - Neda…………….0 |  |
| 107 | | Okola murimu ki, ntegezza nti, murimu ki omukulu gwosinga okukola? | | - Mirimu ejawaaka………….…...1 - Mulimi ………………….…2 - Musomesa………………….....3 - Bizinesi entono-tono……………..4 - Muyizi …….........................5 - Mukugu mu byobulamu…………6 - Ebirara (ki)…………….8 |  |
| 108 | ***Kebeera 104b- Bwaba mufumbo oba ng’abeera ne bba***  Omwami/ ba wo akola murimu ki, ntegezza nti: murimu gwa kika ki gw’asinga okukola? | | - T’akola ……………….…1 - Mulimi ……………..……2 - Musomesa………….…….3 - Bizinesi entono-tono……..4 - Muyizi ………………..….5 - Mukugu mu byobulamu ….6 - Ebirara (ki)…………….….8 | |  |

**EKITUNDU 200; EBINTU EBYEKWASANGANYA KU MAAKA/NJU**

| 201 | Kiki kyemusinga okukozesa nga ekyakiisa mu maaka gamwe? | - Mafuuta……………………... .1 - Manyi ga njuba……….……….2 - Masanyalaze…………….…….3 - Ebirara (ki)……………………4 | |  |
| --- | --- | --- | --- | --- |
| 202 | Wetegereze ekika kyenyumba  Wandiika byolabye. | - Yankalakalira dala……………….1 - Yankalakalira ………2 - Si-Yankalakalira ………….3 | |  |
| 203 | Wetegereze byebaasinga okukozeesa ngabazimba wansi munyumba.  Wandiika byolaba. | | - Wansi tebakolawo/ taaka. . . . . . . . . 1 - Wansi bakolawo/ cement …………2 - Ebirara (Ki)……………………….3 |  |
| 204 | Wetegereze byebaasinga okukozesa ngabazimba akasolya munyumba.  Wandiika byolaba. | | - Akasulya ka subi…………………..1 - Akasulya kankalakalira okugeza amabaati, tiles ne asbestos……..2 - Ebirara (ki) . . . . . …..……….…3 |  |
| 205 | Wetegereze byebaasinga okukozesa ngabazimba ebisenge wabweru.  Wandiika byolaba.  **Ssaza kimu kyoka** | | **Ebisenge sibyankalakalira**   - Emiiti n’ettaka . . . . . . …... . . . . 21 - Amayinja n’ettaka . . . . . . . . . . . . . . 22 - Amatafali agatali mokye n’ettaka. . . ..23 - Plywood . . . . . . . . . . . ….. . . . . . . . 24 - Bibox . . . . . . . . . . . . . . . ………. . . 25 - Embawo enkozeseko . . . . ... . . . . . . . 26 - amatafali agatali mokye ne plaster . . .27 - Amatafali amokye n’ettaka . . . . . . . . .28   **Ebisenge byankalakalira**   - Cementi . . . . . . . . . . . . . . . . . . 31 - Amayinja ne lime/ cementi . . . . . .32 - Amatafali amokye ne cementi . . . . 33 - Buloka za Cement . . . . . . . . . . . . 34 - Amatafali agatali mokye ne cementi .35 - Embaawo. . . . …………………… ..36 - Ebirara (Ki)..……………….……...96 |  |
| 206 | Mu nyumba yamwe/ yo mulimu ebintu bino wamanga:  a) Amasanyalaze/ solar?  b) radio?  c) TV?  d) E simu yokumeza?  e) Computer?  f) Firigi?  g) Radio eyimbisa tape/  CD oba DVD?  h) Meza?  i) Entebe?  j) Sofa set?  k) Ekitanda?  l) Kabada?  m) Sawa yo kukisenge? | | \|  \| **Neda** \| **Yee** \| \| --- \| --- \| --- \| \| a) Amasanyalaze/ solar… \| **0** \| **1** \| \| b) radio……………….... \| **0** \| **1** \| \| c) TV………………… \| **0** \| **1** \| \| d) E simu yokumeza…… \| **0** \| **1** \| \| e) Computer…………… \| **0** \| **1** \| \| f) Firigi…….........……. \| **0** \| **1** \| \| g) Radio eyimbisa tape/ CD oba DVD………………… \| **0** \| **1** \| \| h) Meza………………… \| **0** \| **1** \| \| i) Entebe………………… \| **0** \| **1** \| \| j) Sofa set………………… \| **0** \| **1** \| \| k) Ekitanda…………….. \| **0** \| **1** \| \| l) Kabada……………… \| **0** \| **1** \| \| m) Sawa yo kukisenge… \| **0** \| **1** \| |  |
| 207 | \| Eliyo omuntu yena munyumba eno alina bino wamanga? \| \| --- \| \| a)Sawa yokumukono? \| \| b)Esimu yomungalo? \| \| c)Akagaali? \| \| d) Pikipiki oba sukuta? \| \| e) Ekigali ekisikibwa  ebisolo? \| \| f) Motoka (Kabuyonjo oba etika)? \| \| g) Elyato elye egine? \| \| h) Elyato elitari lya engine? \| | | \|  \| **Neda** \| **Yee** \| \| --- \| --- \| --- \| \| a)Sawa yokumukono……… \| **0** \| **1** \| \| b)Esimu yomungalo………. \| **0** \| **1** \| \| c)Akagaali………………….. \| **0** \| **1** \| \| d) Pikipiki oba sukuta…….. \| **0** \| **1** \| \| e) Ekigali ekisikibwa ebisolo.. \| **0** \| **1** \| \| f) Motoka (Kabuyonjo oba etika) \| **0** \| **1** \| \| g) Elyato elye egine…….. \| **0** \| **1** \| \| h) Elyato elitari lya engine .. \| **0** \| **1** \| |  |

| 208 | Mu nyumba eno mulimu ekisoro oba ekinyonyi kyona ekirudibwa? | - No………………………………..0 - Yes……………………………….1 | **Oba Neda, buuka ogende ku 210** |
| --- | --- | --- | --- |
| 209 | Mbulira omuwendo gwebisoro oba ebinyonyi bino wamanga ebirudibwa mu nyumba eno?   \| a)Ente enansi/ Enganda \| \| --- \| \| b)Ente enzungu oba maleto \| \| c)Edogoyi \| \| d)Enbuzi \| \| e)Endiga \| \| f) Enkooko ne binyonyi ebirara \| \| g)Embizi \| | \| a)Ente enansi/ Enganda… \|  \|  \| \| --- \| --- \| --- \| \| b)Ente enzungu oba maleto \|  \|  \| \| c)Edogoyi \|  \|  \| \| d)Enbuzi……………………. \|  \|  \| \| e)Endiga……………… \|  \|  \| \| f) Enkooko ne binyonyi ebirara \|  \|  \| \| g)Embizi………………… \|  \|  \| |  |
| 210 | Eliyo omuntu yena munyumba eno alina etaka nga bali/ muli limirako? | - Neda………………………………..0 - Yee……………………………….1 | **Oba Neda, buuka ogende ku 212** |
| 211 | Mugate gwa eka meka awamu abantu bo munyumba eno zebarina okurimirwa? | \| Eka \|  \|  \|  \|  \| \| --- \| --- \| --- \| --- \| --- \| |  |
| 212 | Eliyo omuntu yena munyumba eno alina etaka nga tebali/ temuli limirako? | - Neda………………………………..0 - Yee……………………………….1 |  |

**EKITUNDU 300; EBIKWATA KUKUZAALA**

| 301 | Kati nssaba nkubuuze ebibuuzo ebikwatagana ku by’okwegatta. Era nkukakasa nti byona byonaddamu bija kutwalibwa nga byakyama nyo tebijja kwasanguzibwa ew’omuntu yena. Bwetutuka ku kibuuzo kyona ky’otayagala kuddamu, ontegezzako awo netugenda mu masso ne kiddirira. Wali onyumizako embozi ey’ekyama? | | | - Yee…………..1 - Neda………...…0 | | | | **Oba neda, buuka ogende ku 401** | |
| --- | --- | --- | --- | --- | --- | --- | --- | --- | --- |
| 301a | | | Mu bulamu bwo bwona, abaganzi bameka b’obade nabo? | |  | | |  | |
| 302 | | | Walina emyaka emeka we wasokera ddala okunyumya embozi ey’ekyama? | | emyaka | | |  | |
| 302a | **Kebeera 301. Oba Yee,**  Olina abaana? | | | | | | - Yee……….…..1 - Neda……………0 | **Oba Neda, buuka ogende ku 305** | |
| 303 | Olina abaana bameka omugatte/ awamu? | | | | | |  |  |  |
| 304 | ***Kebeera Oba Yee: Eyettabye mu musomo alina abaana abali wansi w’emyaka ettano (5)? Oba Yee***  We wali olubuuto, olubuuto olwo olusembyeyo, wagendako mu kifo ky’ebyobulamu okeberebwa? | | | | | | - Neda………..……0 - Yee…………….1 |  |  |
| 305 | Olinayo abaana ab’obuwala (bawala bo bozaala oba abawala bolabirira) nga bali wakati w’emyaka 10 paka ku 17.  Oba Yee, bali bameka? | | | | | |  |  |  |
| 307 | Gwe, oba omwagalwa wo, waliwo kyemukola kati oba mulina enkola yona gyemukozesa okulindako oba okuziyiza okufuna lubuuto? | | | | | | - Yee……….…..1 - Neda……………0 |  | |
| 308 | | Nkola ki gyemukozesa?  **Wandiika byona ebyogendwa** | | | | - Okusala enseke zo omukyala-1 - Okusala obuseke bwo mwami. . 2 - Akaweeta . . . . . …. . . . …….. . . 3 - Empiiso za famile…….. …….. . 4 - Kapisozi zo kukibega-bega .…. . 5 - Obukelenda bwa famile…... . . . 6 - Kalimpitawa za bami ….….... . . 7 - Kalimpitawa za bakyala…….. . 8 - Famile eza kazigizigi…………. 9 - Akanyere ko mukiwato……...10 - Okuyonsa……………………11 - Enaku zotafuna lubuto.. . . . . . .12 - Okumalira wabwelu . . . ... . . . . .13 - Ekirara(kiiki)……………… . . . 14 | |  | |

| 309 | ***Kebeera 104a- Bwaba mufumbo oba nga abeera ne bba,***  Omwami wo alinayo abakyala abalala oba; abeera n’abakyala abalala nga betwala nga abafumbo? | | - Yee……………..1 - Neda……………….0 | |  | |
| --- | --- | --- | --- | --- | --- | --- |
| 310 | Nga nawe webazze, bwogatta, Omwami wo alina abakyala oba abaganzi bameka? | |  | |  | |
| 311 | Mirundu emeka gy’olabye omusawo webyobulamu omutendeka mu myezi mukaaga egiyise? |  | |  | |  |

**EKITUNDU 400: OKUMANYA KU KOKOLO W’OMUMWA GWA NABAANA**

| **NO.** | **EBIBUUZO N’EBISEGEJJA** | **CODING CATEGORIES** | **BUUKA** |
| --- | --- | --- | --- |
| 401 | Kati gyagala kw’ogera ku kokolo w’omumwa gwa nabaana.  Wali owuliddeko obulwadde bwebayita kokolo w’omumwa gwa nabaana? | - Neda………...0 - Yee……….1 |  |
| 402 | Wa/ ludawa gye wasoka okuyiga ku kokolo w’omumwa gwa nabaana? | - Epapula za maulire ne magazine………....1 - Radio ………………..………..……...…2 - Bipande ebyaka………………….……..3 - Boluganda, bemikwano na bemililano….4 - Obutabo……………………..………….5 - Ebipande ne obubaka obuwandike……..6 - Abakozi bobyobulamu……..………....7 - Banadini………………………….…….8 - Abasomesa………………………..…….9 - Ekilara (kiiki) ………………….…….10 |  |
| 403 | ***Kebeera 303. Oba Yee,***  Bwe wagendako okukebereebwa nga oli lubuuto, wafunayo obubaka bwona obwekulusanya ne kokolo w’omumwa gwa nabaana okuva e w’omusawo w’ebyobulamu? | - Neda………..……0 - Yee…………….1 |  |
| 404 | Mu kulabakwo, ani ali mumatigga/ mukabi ko kufuna kokolo w’omumwa gwa nabaana?  Omwami, omukyala oba omuntu yena? | - Abasajja……………………...1 - Abakazzi……………………2 - Abasajja n’abakazzi bombi……..3 - Abalala (baani) ………….10 |  |
| 405 | Wali owulideyo omuntu ng’alina kokolo w’omumwa gwa nabaana? | - Yee………………….……..1 - Neda…………………………0 |  |
| 406 | Ani asobola okusasanya akawuka akaleeta kokolo w’omumwa gwa nabaana?  Omwami, omukyala oba omuntu yena? | - Abasajja…………………………1 - Abakazzi………………………2 - Abasajja n’abakazzi bombi ………..3 - Simanyi………………......4 - Abalala (bani) ……………….5 |  |
| 407 | Omanyi engeri akawuka ka HPV akavirako kokolo w’omumwa gwa nabaana bwe ka saasana/ bakafunnibwa?  Oba Yee,  Mu ngeri ki akawuka akavirako kokolo w’omumwa gwa nabaaana gye kasasana?  ***(Kiriiza okudamu okwedirigana oba buliliza ebilara*)** | - Okwegata nga tolina kapira…………1 - Okufumitibwa ekitu ekyoji………….2 - Omusikira mumusayi……………..….3 - Amaazi agava mumubirir nga olugezi..3 - Simanyi……………………………..4 - Ekilara (kiiki)……………….………...5 |  |

| 408 | Omanyi obubonero n’ebiraga obulwadde bwa kokolo w’omumwa gwa nabaana?  Oba Yee,  Bubonero n’ebiraga biiki nti omuntu alina obulwadde bwa koolo w’omumwa gwa nabaana?  ***(Kiriiza okudamu okwedirigana oba buliliza ebilara)*** | - Obulumi mu bukyala nga wegata………1 - Okuva omusayi mukyala mu bisela ebitali bya mweezi……………..…………...2 - Okuva mubukyala omusayi nga wakoma okuzala……………………………….....3 - Okuva mubukyala omusayi nga omaze okwegata…………………………….…...4 - Okubundula amazi agawunya obubi….…5 - Okulumizibwa wansi wo lubuto…………6 - Simanyi……………………………….7 - Ebirala (biiki)……………….………...8 |  |
| --- | --- | --- | --- |
| 409 | Mu ndowoza yo, neyiisa/mpiisa ki ezongera amatigga/ akabi g/k’okufuuna akawuka akavirako kokolo w’omumwa gwa nabaana?  **(*Kiriiza okudamu kungi*)** | - Okufuweta sigara………………….1 - Okwegata nga okyali muto…………2 - Okwegata na abantu abangi…..………3 - Okukosebwa edwade ezekikaba………4 - Okuzala abaana abangi………………..5 - Simanyi……………………………6 - Ebirala (Biiki)…………………………7 |  |
| 410 | Wa engeri ezenjawulo eziziyiza kokolo w’omumwa gwa nabaana.  **(*Kiriiza okudamu kungi*)** | - Okwekebeza bulijo………………………1 - Okugemesa akuwuka akalete koolo w’omumwa gwa nabaana …………….2 - Kozesa condom………………………..3 - Okuvira daala kubyokwegata………….4 - kokolo w’omumwa gwa nabaana ajanjabibwa mu dwaliro nga bamulabye nga ta nasajuka/ bukyali…………………5 - kokolo w’omumwa gwa nabaana aziyizibwa nga omusawo akebede ebitundu byo ebyekyama……………………….…..6 - Simanyi………………………………7 - Ebirala (Biiki)……………………….…8 |  |
| 411 | Mu ndowoza yo, kokolo w’omumwa gwa nabaana awonyezebwa/ avumbulwa? | - Neda………………..………..0 - Yee……………….……….1 |  |
| 412 | Kiyinzika kitya omukazzi asangiidwa okuba ne kokolo w’omumwa gw nabaana okubera n’emikiisa miigi egy’okuwona: nga bamumuzudemu nga obudde buyiise/ yasajuka, nga bumumuzudemu mangu, e ssawa yona gye bamumulabamu, oba tomanyi? | - nga bamumuzudemu nga obudde buyiise ……………….1 - nga bumumuzudemu mangu ……………..2 - Simanyi……………………….3 - Ebirala (Biiki)…………………….4 |  |
| 413 | Omanyi engeri kokolo w’omumwa gwa nabaana bw’ajjanjabibwa?  Oba Yee,  Mu ngeri ki ze bajjanjabamu kokolo w’omumwa gwa nabaana?  **(*Kiriiza okudamu kungi*)** | - Okuteka ku kyoma ekikalilira kansa…1 - Kokolo w’omumwa gwa nabaana awonyezebwa mu ddwaliro nga azulidwa mangu ……………....2 - Okulaba omusawo w’ekinansi…...3 - Okukozesa edagala lyekinansi………4 - Simanyi………………......5 - Ebirala (Biiki)……...………6 |  |
| 413a | **Omugatte gwe byo byamanyi ku kokolo w’omumwa gwa nabaana**  ***Wajja kujjuzibwamu oluvanyuma lw’okukunganya okudamu/ ebirowoozo*** |  |  |
| 414 | Mu ndowoza yo, obulwade bwa kokolo w’omumwa gwa nabaana aluma nyo?  Aluma nyo ekyenkomeredde, aluma mu nyo, talumiira ddala, oba tomanyi? | - Aluma nyo ekyekomeredde…………1 - Aluma mu…...2 - T’alumira ddala……...3 - Simanyi…………..4 |  |

**EKITUNDU 500; OKWEKEBEZE KWA** KOKOLO W’OMMWA GWA NABAANA

| 501 | Omuntu asobola atya okumanya oba alina kokolo w’ommwa gwa nabaana? | - Nga agenze o kwekebeza kokolo w’omumwa gwa nabaana………………1 - Simanyi…………………………………2 - Ebirala (Biiki)………………………..…3 |  |
| --- | --- | --- | --- |
| 502 | Kati nsaba kwogera ku kwekebeza kokolo w’omumwa gw nabaana.  Wali owuliddeko ku kwebebeza kokolo w’omumwa gw nabaana? | - Neda……………..0 - Yee……………..1 |  |
| 503 | Wasoka kuyiga otya ku kwekebeza kokolo w’omumwa gwa nabaana? | - Epapula za maulire ne magazine………....1 - Radio ………………..………..……...…2 - Bipande ebyaka………………….……..3 - Boluganda, bemikwano na bemililano….4 - Obutabo……………………..………….5 - Ebipande ne obubaka obuwandike……..6 - Abakozi bobyobulamu……..………....7 - Banadini………………………….…….8 - Abasomesa………………………..…….9 - Ekilara (kiiki) ………………….…….10 |  |
| 504 | Mu kulaba kwo, kirungi omukyala okugenda okwekebeza kokolo w’omumwa gwa nabaana mu bulamu bwe bwona? | - Yee………………………….1 - Neda ………………………….0 |  |
| 504a | Mu kulaba kwo, osengeka otya omugaso gw’omukyala okwetanira okukeberwa kokolo w’omumwa gwa nabaana mu bulamu bwe bwona: kya mugaso nyo, kya mugaso, si kyamugaso, si kyamugaso nakamu, oba tomanyi? | - Kya mugaso nyo ………………1 - Kya mugaso ……………………2 - Si kyamugaso…………………3 - Si kyamugaso nakamu …………...4 - Simanyi…………………..5 |  |
| 505 | Mu kulaba kwo, ani atekeddwa okwekebeza kokolo w’omumwa gwa nabaana: omuntu yena, ow’ebyobulamu omutendeke, omusawo w’ekinansi, oba tomanyi? | - Omuntu yena ……………………1 - Ow’ebyobulamu omutendeke ………..2 - Omusawo w’ekinansi ………………3 - Simanyi……………………4 - Abalala (baani)………………….5 |  |
| 506 | Ku kitundu ki eky’omubiri we okukebeera kokolo w’omumwa gwa nabaana: ekitundu kyona eky’omubiri, ebitundu by’omukyala eby’ekyama, oba tomanyi? | - Ekitundu kyona eky’omubiri ……………1 - Ebitundu by’omukyala eby’ekyama ……..2 - Simanyi…………………………….…….3 - Ebirala (biki)…………………..………..4 |  |
| 507 | Mu kulaba kwo, mu myaka ki egy’obukazi okukeberebwa kokolo w’omumwa gwa nabaana eggisanidde: omwaka gwona, wansi w’emyaka 25, wakati w’emyaka 25-49, wagulu w’emyaka 49 oba tomanyi? | - Omwaka gwona ………………………….1 - Wansi w’emyaka 25……………..……….2 - Wakati w’emyaka 25-49…………………..3 - Wagulu w’emyaka 49 …………………..4 - Simanyi………………………………..….5 |  |
| **507a** | **Omugatte gw’okumanya,**  ***Wajja kujjuzibwamu oluvanyuma lw’okukunganya amawulire/ ebiroowozo*** |  |  |

| NO. | **EBIBUUZO N’EBISEGEJJA** | **EKYOKULANDAKO** | **BUUKA** |
| --- | --- | --- | --- |
| 508 | Omanyi ekiifo kyona abantu we basobola okugenda okwekebeza kokolo w’omumwa gwa nabaana? | - Neda------------0 - Yee-------------1 |  |
| 509 | Abantu basobola kugenda wa okwekebeza kokolo w’omumwa gwa nabaana?  **Elinya lye kiifo……………** | - Eddwaliro lya governmenti--1 - Eddwaliro ly’obwananyini---2 - Awalara (wa)-------------------3 |  |

| 509b | ***kebeera 509, Oba Yee***  Wa ttambula kwenkana ki **(mu ssawa/obuwanvu**) okuva awaaka okutuka ku kiifo aw’akebeererwa? *soyasoya era owandiike e ssawa nga zawukana ku buwanvu* | /  E ssawa / Obuwanvu |  |
| --- | --- | --- | --- |
| 510 | Wali w’ekebeezako ku kokolo w’omumwa gwa nabaana? | - Neda--------0 - Yee---------1 | **Oba Neda, buuka ogende ku 512** |
| 511 | Wa jje bakukebelera kokolo w’omumwa gwa nabaana: Ddwaliro ely’obwananyini, e lya governmenti oba ekiifo eky’ebyobulamu ekirala? | - Ddwaliro ely’obwananyini -----------------1 - Ddwaliro elya governmenti ----------2 - Awalara (Wa)-----------------3 |  |
| 511a | Kiki e kyakuletera/ e kyakusikiriza/e kyakuvirako okugende okwekebeza? | - Okwagala okuziyiza enddwadde-----------------------1 - Okutya enyo okulwara----------------------------------2 - Okufuna olukusaokuva e w’omwami------------------3 - Oluvanyuma lw’okufuna okulumizibwa wansi mu lubuuto-----4 - Oluvanyuma lw’okubeera nga nvamu ekivundu ekibi mu bitundu eby’ekyama----------------------------------5 - Oluvanyuma lw’o kufuna obulumi mu bungo nga nyumya emboozi ey’ekikulu-----------------------------6 - Oluvanyuma lw’okufuna ekikulukuto mu mbugo--7 - Okubeera n’owoluganda alumizibwa kokolo-----8 - Ebirala (Biiki)-------------------------------------9 |  |
| 512 | Mu kulowoza kwo, bwoba oweredwa omukisa, wali kiriza okukeberwa kokolo w’omumwa gwa nabaana?  Oba Yee, wali ssengesse otya okukiriza kwo?  Wewawo yee, ndowoza yee, ndowoza neda, wewawo neda, oba tomanyi? | - Wewawo yee ------------------1 - Ndowoza yee------------------2 - Ndowoza neda----------------3 - Wewawo neda-----------------4 - Simanyi------------------------5 | **Buuka ogende ku 513** |
| 513 | Ani gwe waliyagadde o kukebeera kokolo w’omumwa gwa nabaana? Omusajja, owebyobulamu omukyala, oba tomanyi? | - Omusawo omusajja --------------------------1 - Omusawo omukyala --------------------------2 - Bonna oba musawo musajja ne bwaba mukyala----3 |  |
| 514 | **Kebeera 510. Oba neda,**  Lwaki tewekebeza? | - Tewali budde-------------------------------------------1 - Ntya PAP test (nga ogasekko n’obulumi)----------2 - N’ettaga obudde obulala n’owebyobulamu--------3 - Okwekebeza kwa busere nyo-------------------------4 - Okwekebeza kwa nsonyi nyo------------------------5 - Olugendo lwa wala nyo okutambula------------------6 - Bula lya ntambula---------------------------------------7 - Ebirala (Biiki)-----------------------------------------8 |  |

**EKITUNDU 600: HPV VACCINATION**

| **No.** | **EBIBUUZO N’EBISEGEJJA** | **CODING CATEGORIES** | **BUUKA** |
| --- | --- | --- | --- |
| 601 | Kati jjagala/ nsaba twogere ku kugema kw’abawala abali wakati w’emyaka 10 ne 12 okubatangira okufuna HPV(akawuka akavirako kokolo w’omumwa gwa nabaana)  Wali owulidde ku kugemesa kwa HPV (akawuka akaviraka kokolo w’omumwa gwa nabaana)? | - Yee--------1 - Neda---------0 |  |
| 602 | Olwasoka, wawulira otya ku kugemesa kwa kokolo w’omumwa gwa nabaana? | - Epapula za maulire ne magazine………....1 - Radio ………………..………..……...…2 - Bipande ebyaka………………….……..3 - Boluganda, bemikwano na bemililano….4 - Obutabo……………………..………….5 - Ebipande n’empapula endala zebakubyeko ebigambo-6 - Abakozi be byobulamu-----------------------------------7 - Abakulu ba abanaddini ----------------------------------8 - Abasomesa--------------------------------------------------9 - Abarala (Baani)----------------------------------------10 |  |
| 603 | Mu tuuluba ki e ly’obuwala gye bagemeramu eddagala eriziyiza akawuka akavirako kokolo w’omumwa gwa nabaana : Ku muwala yena, abawala abato aba nyumizaako akabozi k’ekikulu, abawala abato abatanyumyaako akabozi k’ekikulu/mberera oba tomanyi? | - Omuwala yena ----------------------------------------1 - abawala abato aba nyumizaako akabozi k’ekikulu 2 - abawala abato abatanyumyaako akabozi k’ekikulu/ mberera ------------------------------------- -3 - Simanyi----------------------------------------4 - Ebirala (Biiki)-------------------------------------5 |  |
| 603a | Mu kulaba kwo, Mu tuuluba lya myaka ki egy’obuwala gye bagemeramu akawuka akavirako kokolo w’omumwa gwa nabaana | - Omwaka gwona----------------------------1 - Wansi w’emyaka 9 ---------------------2 - Wakati w’emyaka 9-14 --------------------------3 - Wagulu w’emyaka 14 ---------------------4 - Simanyi-------------------------5 - Ebirala (biiki)----------------------6 |  |
| 604a | Mu kulaba kwo, olowoza nti okugemesa eddagala eriziyiza akawuka akavirako kokolo w’omumwa gwa nabaana kw’amugaso eri abawala oba muwala wo? | - Yee------------------------------------1 - Neda-------------------------------------2 |  |
| 604b | Mu kulaba kwo, osengeka otya ebirungi by’okwegemesa eddagala eriziyiza akawuka akavirako kokolo w’omumwa gwa nabaana ku bawala oba bawala bo? | - Kirungi nyo----------------------1 - Kirungi----------------------------2 - Si kirungi------------------------3 - Si kirungi nakamu-----------------4 - Simanyi--------------------------5 |  |
| 604c | Mu kulaba kwo, olowaza nti kikulu nyo okugema abawala abato okulwanyisa akawuka akavirako kokolo w’omumwa gwa nabaana | - Yee -----------------1 - Neda ------------------0 |  |
| 604d | Olowoza lwaki kikulu abawala abato okwegemesa eddagala er-iziyiza akawuka akavirako kok-olo w’omumwa gwa nabaana ? | - Ki ziyiiza abawala abato obutakwatiba kokolo w’omumwa gwa nabaana gyebujja------------------1 - Simanyi--------------------------------------------2 - Ebirala (Biiki)----------------------------------------3 |  |
| 605 | Mu kulaba kwo, ani alina okugema/okuwa eddagala eriziyiza akawuka akavirako kokolo w’omumwa gwa nabaana? | - Musawo w’ekinansi------------------------------------1 - Ow’ebyobulamu mukyalo omutendenke (VHTs)--2 - Ow’ebyobulamu omutendeke-------------------------3 - Omuntu yena--------------------------------------------4 - Abalala (Baani)----------------------------------------5 |  |
| 606 | Omanyi ekiifo kyona we bagemera eddagala eriziyiza akawuka akavirako kokolo w’omumwa gwa nabaana?  **Oba Yee, wa erinya ly’ekiifo**  **-----------** | - Yee-----------------------------1 - Neda------------------------------0 |  |
| 606a | Waliwo buwanvu ki bwoba otambudde ku bigere **(ssawa/ buwanvu)** okuva ewaka okutuka mu kiifo we bagemera eddagala eriziyiza akawuka akavirako kokolo w’omumwa gwa nabaana? *Soyasoya owandiike e ssawa zoka okwawukana ku buwanvu bwe kiifo.* | /  E ssawa / Obuwanvu |  |
| 608 | Omuwala agemebwa emirudi emeka okumalayo dozi ye eddagala eriziyiza akawuka akavirako kokolo w’omumwa gwa nabaana: Gumu, ebiri, oba ebirala? | - Gumu---------------1 - Ebiri-----------------2 - Simanyo------------3 - Ebirala (Biiki)-----4 |  |
| 609 | Mu ndaba yo, wayitawo banga ki okumugema omulundi ogwokubiri: e myezi mukaaga (6), Omwaka gumu oba tomanyi? | - E myezi mukaaga --------------1 - Omwaka gumu ------------------2 - Simanyi----------3 - Ebirala (ddi)--------4 |  |
| 609a | **Omugatte gw’okumanya ku** eddagala eriziyiza akawuka akavirako kokolo w’omumwa gwa nabaana?**,**  ***Wajja kujjuzibwamu oluvanyuma lwa okukunganya ebirowoozo*** |  |  |

**Ngenda kukubuuza ebibuuzo ebidako. Ddamu buli kibuuzo nti Yee, Neda oba simanyi**

|  | Ebibuuzo | Yee……1 | Neda.2 | Simanyi..3 |
| --- | --- | --- | --- | --- |
| 610 | Wali tuute bawala bo okugemessebwa? |  |  |  |
| 611 | Omwami/muganzi wo yali kiriiza/waggidde ekya bawala bamwa okugemesebwa eddagala eriziyiza akawuka akavirako kokolo w’omumwa gwa nabaana? |  |  |  |
| 612 | Mu kulaba kwo, olina obusobozi okutukirira omukozi w’ebyobulamu omutendeke okugemesa bawala bo? |  |  |  |

| 613 | Kati nsaba/njagala twogere ku bintu ebyobulabe ebyekulusanya ne kokolo w’omumwa gwa nabaana.  Ofuweeta ku sigara? | - Yee---------------1 - Neda----------------0 |  |
| --- | --- | --- | --- |
| 613a | Onywa omwenge? | - Yee----------------1 - Neda-----------------0 |  |

**Kebeera 305, Oba Yee**

**Kakati, nsaba kuwandiika amanya awamu n’ebilara ebye kulusanyiza/ebikwatagana n’okugemesa kwa eddagala eriziyiza akawuka akavirako kokolo w’omumwa gwa nabaana**? **okwa…. (number (kebeera 305)) abawala okutandikira ku myaka 10 okutuuka ku myaka 17.**

***Wandiika amanya g’abawala bona mu kabox 615 abali wakati w’emyaka 10-17***

| **614** | **615** | **616** | **617** | **617a** | **618** | **619** | **620** | **621** | **622** | **623** | **…………………………………………..Emmeza yeyongera yo** |
| --- | --- | --- | --- | --- | --- | --- | --- | --- | --- | --- | --- |
|  |  |  |  |  |  | ***Kebeera 618***  ***Oba Yee,*** |  |  | ***Kebeera 620***  ***Oba Yee*** | ***Kebeera 620 Oba Yee*** |  |
| Namba ya maama | Namba yo mwana  **Amanya** | Emyaka egyo mwana | Wakumeka ku maama | …(amanya) Omyita otya?  **A=Gwe amuz-alira dala..1**  **B=Ekirala (otya)...2** | Is…(Amanya) Asoma kati?  **A=Yee..1**  **B=Neda….0** | Essomero lya kika ki?  **A=Governmenti..1**  **B=Bwananyini..2**  **C=Ebirala**  **(Biiki)..3** | (Amanya) ya gemese-bwa okwe-wala HPV –Okukebeera oba nga wekiri  **A=Yee--1**  **B=Neda--0** | Mirundi emeka? **Kebeera ku kada**  **A=Gumu B=Ebiri C=Simanyi** | Bamugemera luda wa?  **A=Ku sso-mero..1**  **B=Awaaka2**  **C=Mu kiifo ky’ebyobulamu….3** | Ani yassalawo ...(amanya) bwe ya gemesebwa?  **A=Mukyala-1**  **B=muganzi-2**  **C=Bombi-3**  **D=Tewali-4**  **E=Abalara (baani)…..5** |  |
|  |  |  |  |  |  |  | **Oba Neda, genda ku 625** |  |  |  |  |
|  | 01 |  |  |  |  |  |  |  |  |  |  |
|  | 02 |  |  |  |  |  |  |  |  |  |  |
|  | 03 |  |  |  |  |  |  |  |  |  |  |
|  | 04 |  |  |  |  |  |  |  |  |  |  |

| **KWOLONDA OKUJUZA Q 625 $ 629**  **A**=Talina kumanyisibwa ku HPV----1  **B**=Muto nyo okugemesebwa--2  **C=**Tali mu bulabe kufuna HPV------------3  **D=**Simanyi ooba edagal si lyabulabe---4  E=Omwana wange ayinza okufuuka omugumba gy’ebujja -5  F=Ntya muwala wange okufuka omwenzi6  G=Sirina wesobola kufuna kugemesebwa--7  H=Simanyi ani gwe nvunana ku biva mu kugemesebwa----------------8  **I=**Ebirala (biiki)---------------------------9  **CODES ZA Q 628**  A=Ya yimirizibwa okugenda ku ssomero olunaku olwo----------------------------------1  B=Yagoba abakozi b’ebyobulamu awaaka2  C= Omwana yaweebwa emiriimu emirala mukiseera ekyo kugemesebwa--------------3  **D=**Omwana yatisiibwa tisiibwa nti okugemesebwa kwalina ebizibu obubivamu----------4  **E=**Ebirala (Biiki)----------------------5   \| **615** \| **624** \| **625** \| **626** \| **627** \| **628** \| **629** \| \| --- \| --- \| --- \| --- \| --- \| --- \| --- \| \|  \| ***Kebeera 620 Oba Yee*** \| **Kebeera 620**  **Oba neda,** \| **Kebeera 620 oba neda,** \| **Kebeera 620 Oba neda,** \| **Kebeea 620 oba neda,** \| **Kebeera 620 oba neda,** \| \| Namba yo mwana  **Amanya** \| Obuyambi bwa ngeri ki omwami wo bweyakuwa …(amanya) okugemesebwa?  **A=Okubulirira..1**  **B=Entambula…..2**  **C=Ebirala (biiki)…….3** \| Nsonga ki ezaganyisa okugemesebwa kwa…(amanya)?  **Kebeera kudyo mu kasonda ofuune ekyokujuzawo** \| Wali kiriiza …. (amanya) okugemesebwa mu myezi mukaaga ejjomumaso bwoba oweredwa omukisa?  **A=Yee…1**  **B=Neda….0** \| Omwami wo yaganako okugemesebwa kwa..(amanya)  **A=yee.1**  **B=Neda..0** \| Yagana atya okugemesebwa kwa..(amanya) \| Olwa…(amanya) songa ki ezawebwa okugana okugemesebwa kuno? \| \|  \|  \|  \| ***Kebeera 620 Oba Yee, buuka ogende ku 701*** \|  \|  \|  \| \| **01** \|  \|  \|  \|  \|  \|  \| \| **02** \|  \|  \|  \|  \|  \|  \| \| **03** \|  \|  \|  \|  \|  \|  \| \| **04** \|  \|  \|  \|  \|  \|  \| |
| --- | --- | --- | --- | --- | --- | --- | --- | --- | --- | --- | --- | --- | --- | --- | --- | --- | --- | --- | --- | --- | --- | --- | --- | --- | --- | --- | --- | --- | --- | --- | --- | --- | --- | --- | --- | --- | --- | --- | --- | --- | --- | --- | --- | --- | --- | --- | --- | --- | --- | --- | --- | --- | --- | --- | --- | --- |

**EKITUNDU 700; ESONGA EBYEKULUSANYIZA KU OMWAMI/ OMUGANZI**

| **NO.** | ***EBIBUUZO N’OBUSENGEJJA*** | **EKYOKULONDAKO** | **BUUKA** |
| --- | --- | --- | --- |
| 701 | ***Kebeera -104a- Bwaba nga ali mu bufumbo***  Kati nsaba/njagala twogere ku nsonga ez’ekulusanyiza ku mwami/muganzi wo.  Omwami wo alina emyaka emeka? |  | ***Kebeera 104a bwabera nga taali mu bufumbo bwona, buuka ogende ku nkomerero*** |
| 702 | Omwami wo yasoma paka ku mutindo ki? | - Teyasoma---0 - Primary-----------1 - Secondary--------2 - Tertiary-----------3 - University--------4 |  |
| 703 | Osaba olukuusa buli ly’obeera tonagenda kujjanjabibwa mu ddwaliro? | - Yee-----------------1 - Neda------------------0 |  |
| 704 | Biiki ebikuvirako okusaba olukuusa nga tonagenda kujjajabibwa mu ddwaliro? | - Nga ndi mulwadde-------------------------1 - We bansidika mu ddwaliro ely’ewala ----2 - Ebirala (Biiki)----------------3 |  |
| 704a | Ani attera okusalawo ku byobulamu/bujjanjabi bwo: gwe, omwami/muganzi wo, oba mwembi wamu gwe n’omwami/muganzi wo, oba omuntu omulala? | - Nze-----------------------------------1 - Omwami/muganzi wange --------------------2 - Ffembi wamu nze n’omwami/muganzi wange --3 - Omuntu omulala --------------------------------4 - Abarala (Baani)--------------------------------5 |  |

**OBUVUNANYIZIBWA BW’OMWANI MU KUKEBEERA KOKOLO W’OMUMWA GWA NABAANA**

| 705 | Wali osaabye ko olukuusa e w’omwami wo okugenda okwekebeza kokolo w’omumwa gwa nabaana?? | - Neda--------------0 - Yee------------1 | **Oba neda, buuka ogende e wassemba** |
| --- | --- | --- | --- |
| 706 | We wasaaba olukuusa okugende okukeberebwa, yaddamu atya? | - Teya kikiriiza/ Ya kigaana-------0 - Ya kiriiza------------------1 | ***Oba 0, gende ku 708*** |
| 707 | Bwaba yakiriiza, buyambi ki bweyakuwa/bweyaleeta? (mu by’okukebeera) | - Ensimbi------------------1 - Ssente z’entambula-----2 - Kuzamu manyi----------3 - Okumanyisa---------------4 |  |
| 708 | **Kebeera 706 Oba 0,**  Ssonga ki ezaganyisa ekiteeso?  **Kiriiza okuddamu kungi** | - Tewali budde---------------------------1 - Okutya okunkebera kokolo wo mumwa gwa nabaana (ng’otaddeko obulumi)--2 - Okukeberwa kwa beeyi nyo-----------------3 - Okukeberwa kwa nsonyi------------4 - Waala okutambula wo /okutuuka yo-------5 - Tewali ntambula---------------6 - Ebirala (biiki) -----------------------9 |  |
| 709 | Wakola ki bwe yakugaana okugenda okukeberebwa? | - Na zimula ekiragiiro kye-----------------------1 - Nalinda mpaka weyampa ssente------2 - Ninda anfunireyo ku ssente -3 - Ninda ampe olukuusa olw’okugendayo------4 - Ebirala (biiki)-------------------------------4 |  |

***Webale nyo okwetaba mu kunonyereza kuno .***

***Ekigatidwako 4: Ebibuzo bye kikunsu kya bakyala a byemyaka 25-49***

**Ebibuzo ebitandika**

1. Kiki kyomanyi ku kokolo w’omumwa gwa nabaana? Soyasoya: amanya ag’ekinansi, ekibuleta/kwe buva n’obubonero.

2. Kokolo w’omumwa gwa nabaana akuula/atambula atya?

**Omutwe 1: Okutegera kwa kokolo w’omumwa gwa nabaana mukitudu kino**

3. Kiki ekivirako kokolo w’omumwa gwa nabaana? (Soyasoya ku: ani abufuuna awamu n’ani asinga okubeera mumatinga okubufuuna)

5. Kokolo w’omumwa gwa nabaana alina bubunero ki?

6. Wali osanzeko omuntu alumizibwa kokolo w’omumwa gwa nabaana?

(i)Walaba ki/ Wetegereza ki?

8. Mu ngeri ki kokolo w’omumwa gwa nabaana jje bamujjanjaba? (soyasoya ku ngeri ez’ekinansi, engeri enkugufu/enkugu ne okuyinzika okumuwonya okusinzira ku nkola ezimenyedwa mu kumujjanjaba)

9. Okutuusa kati, kiki ekikoledwawo mu nsi eno ne mu kiifo w’obeera okutangira kokolo w’omumwa gwa nabaana?

**Omutwe 2: Okutangira kokolo w’omumwa gwa nabaana mukitundu kino**

9. kokolo w’omumwa gwa nabaana omwetangira otya?

(i) Ani alina okugemebwa?

(ii) Bantu ki abasinide/ bebakubiliza okwekebeza kokolo w’omumwa gwa nabaana?

10. Okuva ku byowulira mu bakyala mu kitundu kino, bakeberebwa?

(i) Bakyogerako ki?

(ii) Lwaki tebekebeza?

11. Abantu mu kitundu kino bewayo/bagala okutwala bawala babwe okugemebwa okulwanyisa akawuka akaleta kokolo wo mumwa gwa nabaana?

(i) Abasajja bawagiira batya mu kugemessa bawala babwe okuziyiza akawuka akaleta kokolo wo mumwa gwa nabaana?

(ii) Ate bakyala babwe okukeberebwa kokolo w’omumwa gwa nabaana?

12. Kiki abasajja, nga abaami/abaganzi ne bataata kyebalowooza ku:

(i) Kugemesa akawuka akaleta kokolo wo mumwa gwa nabaana ne,

(ii) Okukeberebwa kwa bakyala babwe kokolo w’omumwa gwa nabaana?

**Ebibuuzo ebifundiikira**

12. Mubintu byona bye twogeddeko leero, olina yo ebirala byona eby’okwogera ku kokolo w’omumwa gwa nabaana?

***Ekigatidwako 4: Ebibuzo bye kikunsu kya bawala a byemyaka 10-17***

**Ebibuzo ebitandika**

1. Kiki kyomanyi ku kokolo w’omumwa gwa nabaana? Soyasoya: amanya ag’ekinansi, ekibuleta/kwe buva n’obubonero.

**Omutwe 1: Okutegera kwa kokolo w’omumwa gwa nabaana mu kitundu kino**

1. Kiki ekireeta/ekivirako kokolo w’omumwa gwa nabaana? (Soyasoya ku ani abufuuna ne ani asinga okubera mubulabe okubufuuna)

**Omutwe 2: Okutegera okutangira kokolo w’omumwa gwa nabaana mu kitundu kino**

1. Kokolo w’omumwa gwa nabaana bwaba tatangidwa oba tayanguyibwa kujjajaba mangu, osobola okumbulira kiki ekibeerawo?
2. Kokolo w’omumwa gwa nabaana atangibwa? Ajjanjabibwa atya mu kitundu kino?
3. Atangirwa atya?

Okutegera okutangira kokolo w’omumwa gwa nabaana kisobola okukolebwa mu kugemessa abawala abato?

1. Omanyiyo abawala abato mu kitundu abagemebwa?
2. Babagemera wa?
3. Abawala abato babakiriza okwebuza ku bazadde babwe/ ababalabirira nga tebanagemebwa?
4. Bakitwala batya? (soyasoya taata ne maama)
5. Abawala bona mu ssomero lyo bagemebwa?
6. Songa ki ezaweebwa abo abatagemebwa?

**Ekibuuzo ekifundiikira**

13. Kiki ekirina okukolebwa okwongera omutindo gw’okugemesa akawuka akaleta kokolo wo mumwa gwa nabaana mu bawala abato?

***Ekigatidwako 4: Ebibuzo bye kikunsu kya basaja abafumbo***

**Ebibuzo ebitandika**

1. Kiki abantu mu kitundu kino kye bamanyi ku kokolo w’omumwa gwa nabaana? Soyasoya: amanya ag’ekinansi, ekibuuleta/kwebuva n’obubonero

**Omutwe 1: Okutegera kwa kokolo w’omumwa gwa nabaana mukitundu kino**

3. Kiki ekireeta/ekivirako kokolo w’omumwa gwa nabaana? (Soyasoya ku ani abufuuna ne ani asinga okubera mu bulabe okubufuuna)

5. Kokolo w’omumwa gwa nabaana alina bubunero ki?

6. Wali olabyeko/osanzeko omuntu yena alumizibwa kokolo w’omumwa gwa nabaana?

(i) Walaba kii?

9. Kakano kiki eky’akakolebwa mu kitundu kino okwentangira kokolo w’omumwa gwa nabaana?

**Omutwe 2: Okutangira kokolo w’omumwa gwa nabaana mukitundu kino**

9. Kokolo w’omumwa gwa nabaana omwetangira otya?

(i) Ani alina okugemebwa?

(ii) Ani alina ebisanizo okukeberebwa kokolo w’omumwa gwa nabaana?

10. Okuva ku by’owulira ku bakyala mu kitundu kino, bakeberebwa?

(i) Bakyogerako ki?

(ii) Lwaki tebekebeza?

11. Abantu mu kitundu kino bewayo/bagala okutwala bawala babwe okugemebwa akawuka akaleta kokolo wo mumwa gwa nabaana?

(i) Abasajja bawagiira batya mu kugemessa bawala babwe akawuka akaleta kokolo wo mumwa gwa nabaana?

(ii) Ate bakyala babwe okukeberebwa kokolo w’omumwa gwa nabaana?

12. Kiki abasajja nga abaami/abaganzi ne bataata kyebalowooza ku:;

(i) Kugemesa bawala babwe akawuka akaleta kokolo wo mumwa gwa nabaana,

(ii) Okukeberebwa kokolo w’omumwa gwa nabaana?

**Ebibuuzo ebifundikira**

12. Mu bintu byona bye twogeddeko leero, olina yo ebirala byona eby’okwogera ku kokolo w’omumwa gwa nabaana?

***Ekigatidwako 7: Ebibuzo ebya basawo***

**Ebibuzo ebitandika**

1. Kiki abantu mu kitundu kino kye bamanyi ku kokolo w’omumwa gwa nabaana? Soyasoya: amanya ag’ekinansi, ekibuuleta/kwebuva n’obubonero

**Omutwe 1: Okutegera kwa kokolo w’omumwa gwa nabaana mu kitundu kino**

3. Abantu mu kitundu kino bategera ki nga ekireeta/ekivirako kokolo w’omumwa gwa nabaana?

5. Abatuuze mu kitundu kino basobola okulaba/ okutegera obubonero bwa kokolo w’omumwa gwa nabaana? (soyasoya ku mitendera jj’okubulaabirako)

**Omutwe 2: Okutegera okutangira kokolo w’omumwa gwa nabaana mukitundu kino**

6. Abantu b’omu kitundu bajjanjaba/bavunika batya kokolo w’omumwa gwa nabaana?

7. Abantu b’omu kitundu ba jumbidde batya ku bintu by’okwetangira kokolo w’omumwa gwa nabaana?

8. Abantu bajja mangu okwekebeza?

9. Mu kulaba kwo, kusomwozebwa ki na biiki ebibasobosobozesa mu kwetangira obulungi kokolo w’omumwa gwa nabaana? (Soyasoya ku kwekebeza n’okugemesebwa)

10. Abasajja bawagira/bayambako mu kutangira kokolo w’omumwa gwa nabaana?

11. Bawagira/bayambyeko batya? (soya soya ku kwekebeza n’okugemesebwa)

**Ebibuuzo ebifundikira**

13. Mu bintu byona bye twogeddeko leero, biiki byoteesa/byoyagala bikolebwe okulaba nti kokolo w’omumwa gwa nabaana atangirwa (ku kwekebeza n’okugemesebwa) kweyongere?

***Ekigatidwako 7: Ebibuzo ebya basomesa***

**Ebibuzo ebitandika**

**Omutwe 1 : Okutegera okutangira kokolo w’omumwa gwa nabaana mukitundu kino**

1. Abantu mu kitundu kino balina ndowooza ki ku kokolo w’omumwa gwa nabaana?

(i) Linya ki ery’ekinansi lyebatuuma kokolo w’omumwa gwa nabaana mu Buganda/ Nakasongola?

(ii) Kiki/ biki ekirowozebwa okubeera obubonero bwa kokolo w’omumwa gwa nabaana?

(iii) Kiki/ biki ekirowozebwa okuleeta/okuvirako kokolo w’omumwa gwa nabaana?

(iv) Ajjajjabwa atya, bamukakanya batya?

**Omutwe 2 : Ebikoledwa okuziyiza**

2. Waliwo entekateka ezo kugemesa HPV eziddukanyizibwa oba ezinaddukanyizibwa ku ssomero lyo?

3. Abaana boona abalina ebisanyizo bagemesebwa okwetangira HPV mu ssomero lyo? (soya soya omuwendo gw’emirundi gye kyakolebwa awamu n’ebbanga kyelyatwala wakati w’okugemesa)

(i) Abawala bona bagemesebwa? Oba neda, lwaki?

4. Ab’omukitundu mw’obera awaamu n’abazadde, bajumbira batya ku ntegekka ey’okugemesa? (Soya soya ku bantu abakiliza nabo abagaana.

(i) Ensonga zabwa ze ziriwa (wezibeera yo)?

5. E kitundu jjobeera ky’akungibwa ng’entegekka z’okugemesebwa tezinatandika? Kyakolebwa kitya?

6. Abaana bakiriizibwa okwebuuzako oba okubulira ku bazadde babwe?

7. Abazadde baddamu/ bakitwala batya? (Soya soya ku bantu abakiliza nabo abagaana)

8. Lwaki abazadde ba; (a) kiriiza oba (b) bagana?

**Ebibuuzo ebifundikira**

9. Singa entegeka eno yeyongerayo mu maaso, biiki ebiriina okukolebwa okwongera omutindo gw’okugemesebwa kw’abawala okwetangira akawuka akaleeta kokolo w’omumwa gwa nabaana?
