# Supplementary material for: Predictors of cervical cancer screening uptake in two districts of Central Uganda
Source: PLoS One. 2020 Dec 3;15(12):e0243281. doi: 10.1371/journal.pone.0243281 (PMC7714132; doi:10.1371/journal.pone.0243281)
Supplement: S1 Questionnaire — (DOCX) [file pone.0243281.s003.docx]

## Questionnaire for predictors of cervical cancer prevention practices

**SURVEY TOOL FOR WOMEN AGE 25-49 YEARS FOR NAKASONGOLA AND WAKISO DISTRICTS**

Identification number__________________________________

Name of interviewer:……………………………….

Signature……………………………………………..Date………………….

**START**

**TIME**

**HOUR**

**MINUTES**

**Interviewer instructions:**

***Place a***  ***in the box of the selected answer(s).***

Do not read responses unless the directions indicate.

**Identifying information**

| **NO.** | **QUESTIONS AND FILTERS** | **CODING CATEGORIES** | **SKIP** |
| --- | --- | --- | --- |
| 100A | Type of residence | - Rural------------0 - Urban------------1 |  |
| 100B | District | - Wakiso------------1 - Nakasongola------2 |  |
| 100C | Division/ Sub county |  |  |
| 100D | Village/ LC/ Ward |  |  |

**SECTION 100: BACKGROUND CHARACTERISTICS**

| **No.** | **QUESTIONS AND FILTERS** | **CODING CATEGORIES** | | | | | **SKIP** |
| --- | --- | --- | --- | --- | --- | --- | --- |
| 101 | In what month and year were you born? | ______/_____  Month/ Year | | | | |  |
| 101a | How old were you at your last birth birthday? |  |  |  |  | Years |  |
| 102 | Have you ever attended school?    **If yes**    What is the highest level of education you have completed? | - No education………….1 - Primary………………..2 - O level…………………3 - A level………………….4 - University……………....5 - Tertiary…………………6 | | | | |  |
| 103 | What is your religion? | - Catholic…….…….…….1 - Protestant ........................2 - Muslim ..………….....… 3  Pentecostal……….…..... 4 - SDA……………...……. 5 - Other (Specify).…...……..6 | | | | |  |
| 104 | What is your tribe? | - Muganda . . . . …………..1 - Munyankole……………. 2 - Musoga . .. ……...….... . . 3 - Mukiga ….…………… . . 4  Muruli . . ……………..…. 5 - Other (Specify) …………...6 | | | | |  |

| 104a | Have you ever married or lived together with a man as if married? |  Yes……………..1  No………………0 | | | | | **If no, skip to 105** |
| --- | --- | --- | --- | --- | --- | --- | --- |
| 104b | What is your marital status now: are you widowed, divorced, or separated? | - Married…………………….1 - Widowed …………………..2 - Divorced……………..……..3 - Separated ………….......……4 | | | | |  |
| 104c | How old were you at first marriage? |  |  |  |  | Years |  |
| 105 | What is the sex of the head of this household? |  Male………….1  Female………..2 | | | | |  |
| 106 | Are you currently engaged in paid work? |  Yes …………..1  No…………….0 | | | | |  |
| 107 | What is your occupation, that is , what kind of work do you mainly do? | - Housewife…………….…...1 - Farmer ………………….…2 - Teacher………………….....3 - Small business……………..4 - Student …….........................5 - Health professional…………6 - Others (Specify)…………….8 | | | | |  |
| 108 | ***Check 104b- If married or cohabiting*** What is the occupation of your spouse, that is, what kind of work does he mainly do? | - Not employed …1 - Farmer …………2 - Teacher………….3 - Small business…..4 - Student ………..….5 - Health professional….6 - Others---------------------8(specify) | | | | |  |

**SECTION 200; HOUSEHOLD CHARACTERISTICS**

| 201 | What is the main source of lighting in your house hold? |    | keresone………………. .1 Solar energy…………….2 |  |
| --- | --- | --- | --- | --- |
|  |  |  | HEP……………….…….3 |  |
|  |  |  | Others (Specify)……………………4 |  |
| 202 | Observe the type of the house Record observation. |    | Permanent……………….1 Semi-permanent…………2 |  |
|  |  |  | Temporary……………….3 |  |
| 203 | Observe main material of the floor of the dwelling.  Record observation. |      | Natural floor. . . . . . . . . . . . 1  Finished floor/ cement ……2  Other (Specify)…………….3 |  |
| 204 | Observe main material of the roof of the dwelling.  Record observation. |    | Natural roofing e.g grass……...1  Finished roofing e.g iron sheets, tiles  & asbestos……………………..2 |  |
|  |  |  | Other (Specify) . . . . . …..……3 |  |

| 205 | Observe main material of the exterior walls of the dwelling.  Record observation.                    **Mark only one** | **Rudimentary walls**   - Poles with mud . . . . . . …... . . . . 21 - Stone with mud . . . . . . . . . . . . . . 22 - Unburnt bricks with mud. . . . . . ..23 - Plywood . . . . . . . . . . . .. . . . . . . . 24 - Cardboard . . . . . . . . . . . . . . . . . . 25 - Reused wood . . . . . . ... . . . . . . . 26 - Unburnt bricks with plaster . . . . .27 - Burnt bricks with mud . . . . . . . . .28   **Finished walls**   - Cement . . . . . . . . . . . . . . . . . . 31 - Stone with lime/cement . . . . . .32 - Burnt bricks with cement . . . . 33 - Cement blocks . . . . . . . . . . . . 34 - Unburnt bricks with cement . .35 - Wood planks/shingles . . . . . ..36 - Other (Specify)..……………...96 | | |  |
| --- | --- | --- | --- | --- | --- |
| 206 | Does your house hold have: a) Electricity?   1. A radio? 2. A television? 3. A non-mobile telephone? 4. A computer? 5. A refrigerator? 6. A cassette/ CD/DVD player? 7. A table? 8. A chair? 9. A sofa set? 10. A bed? 11. A cup board) 12. A clock? | 1. Electricity…………….. 2. A radio………………….. 3. A television………………. 4. A non-mobile telephone… 5. A computer………………. 6. A refrigerator…………… 7. A cassette/ CD/DVD player.. 8. A table…………………….. 9. A chair……………………. 10. A sofa set………………….. 11. A bed……………………... 12. A cup board………………. 13. A clock………………….. | **No**  **0**  **0**  **0**  **0**  **0**  **0**  **0**  **0**  **0**  **0**  **0**  **0**  **0** | **Yes**  **1**  **1**  **1**  **1**  **1**  **1**  **1**  **1**  **1**  **1**  **1**  **1**  **1** |  |
| 207 | Does any member of this house hold own:     1. A watch? 2. A mobile phone? 3. A bicycle? 4. A motor cycle or motor?   scooter   1. An animal cut? 2. car/truck? 3. Boat with a motor?   h)Boat without motor? | 1. A watch…………………… 2. A mobile phone………….. 3. A bicycle………………….. 4. A motor cycle or motor scooter……………………….. 5. An animal cart……………... 6. car/truck……………………. 7. Boat with a motor…………..   h)Boat without motor……….... | **No**    **0**  **0**  **0**    **0**  **0**  **0**  **0**  **0** | **Yes**    **1**  **1**  **1**    **1**  **1**  **1**  **1**  **1** |  |
| 208 | Does this house hold own any livestock, herds, other farm animals, or poultry? | - No………………………………..0 - Yes……………………………….1 | | | If no, skip to 210 |

| 209 | How many of the following animals does this house hold own?  a)Local cattle?  b)Exotic/ cross breed cattle?  c)Horses, donkeys, or mules?  d)Goats?  e)Sheep?  f) Chicken or other poultry?  g)Pigs? | a)Local cattle………………  b)Exotic/ cross breed cattle  c)Horses, donkeys, or mules  d)Goats…………………….  e)Sheep……………………  f) Chicken or other poultry..  g)Pigs……………………… | | | | |  |  |  |  |
| --- | --- | --- | --- | --- | --- | --- | --- | --- | --- | --- |
|  |  |  |  |  |  |  |  |  |  |  |
|  |  |  |  |  |  |  |  |  |  |  |
|  |  |  |  |  |  |  |  |  |  |  |
|  |  |  |  |  |  |  |  |  |  |  |
|  |  |  |  |  |  |  |  |  |  |  |
|  |  |  |  |  |  |  |  |  |  |  |
|  |  |  |  |  |  |  |  | |  |  |
| 210 | Does any member of this household own any agricultural land? | - No………………………………..0 - Yes……………………………….1 | | | | | | |  | If no skip to 212 |
| 211 | How many acres of agricultural land do members of this household own? |  | Acres |  |  |  | |  |  |  |
| 212 | Does any member of this household own any non-agricultural land? | - No………………………………..0 - Yes……………………………….1 | | | | | | |  |  |

**SECTION 300; REPRODUCTION INFORMATION**

| 301 | Now I would like to ask you some questions related to your sexual activity. Let me assure you again that your answers are completely confidential and will not be told to anyone. If we come to any question that you do not want to answer, just let me know and we will go on to the next question.  Have you ever had sex? | |    | | | | Yes…………..1  No………...…0 | | | | **If no, skip to 401** |
| --- | --- | --- | --- | --- | --- | --- | --- | --- | --- | --- | --- |
| 301a |  | In your life time, how many sexual partners have you had? |  |  |  | |  | | | |  |
|  |  |  |  |  | | |  |  |  |  |  |
| 302 |  | How old were you when you first had sex? |  |  |  | | years | | | |  |
|  |  |  |  |  | | |  |  |  |  |  |
| 302a | **Check 301. If yes,**  Do you have children? | | | | | | - Yes……….…..1 - No……………0 | | | | **If no skip to 305** |
| 303 | How many children do you have altogether | | | | | |  |  |  |  |  |
|  |  |  |  |  |  |  |  |  | |  |  |
| 303a | How old were you by the time you gave birth to your first child? | | | | | |  |  |  | Years |  |
|  |  |  |  |  |  |  |  |  | |  |  |
| 303b | **Check 303. if more than one,**  Are these children of the same father?  If no how many men have you fathered with your children? | | | | | |  | | | |  |
|  |  |  |  |  |  |  |  |  |  |  |  |
| 304 | ***Check 301if yes: does the respondent have children under 5 years? If yes***  When you were pregnant with your last pregnancy, did you visit a health facility for antenatal care? | | | | | | - No………..……0 - Yes…………….1 | | | |  |
| 305 | Do you have any girls (your daughters or girls under your care) age 10 to 17 years.  If yes how many? | | | | | |  | | | |  |
|  |  |  |  |  |  |  |  |  |  |  |  |
| 307 | Are you or your partner currently doing something or using any method to delay or avoid getting pregnant? | | | | |    | | | | Yes……….…..1  No……………0 |  |
| 308 | Which method are you using?                  **Record all mentioned** | | | | |                    | | | | Female sterilization……1  Male sterilization……. . 2  IUD . . . . . . . . . …….. . . 3  Injectables……..…….. . 4  Implants . . . . . . . ……. . 5  Pill . . . . . …...…….. . . . 6  Condom …..……….... . . 7  Female condom . …….. . 8  Emergency contraception. 9 Standard days method/moon  beads…………………...10 |  |
|  |  | | | | |  | | | | Lactational amenorrhea  method…………………11 |  |
|  |  | | | | |  | | | | Rhythm method . . . . . . .12 |  |
|  |  | | | | |  | | | | Withdrawal . . . . ... . . . . .13 |  |
|  |  | | | | |  | | | | Other modern method . . . 14 |  |
| 309 | ***Check 104a- If married or cohabiting,*** Does your husband/partner have other wives or does he live with other women as if married? | | | | |    | | | | Yes……………..1 No……………….0 | If no skip to 311 |
| 310 | Including yourself, in total, how many wives or live-in partners does he have? | | | | |  | | | |  |  |
|  |  |  |  |  |  |  | |  |  |  |  |
| 311 | How many times have you seen a trained health worker in the last six months? | | | | |  | | | |  |  |
|  |  |  |  |  |  |  | |  |  |  |  |

**SECTION 400: CERVICAL CANCER KNOWLEDGE AND AWARENESS**

| **NO.** | **QUESTION AND FILTERS** | **CODING CATEGORIES** | **SKIP** |
| --- | --- | --- | --- |
| 401 | Now I would like to talk about cervical cancer.    Have you ever heard of a disease called cervical cancer? | - No………...0 - Yes……….1 |  |
| 402 | Where did you first learn about cervical cancer? | - Newspapers and magazine………....1 - Radio …………………………...…2 - Billboards…………………………..3 - Family, friends, neighbors and colleagues.4 - Brochures…………………………….5 - posters and other printed materials…..6 - Health workers……………………....7 - Religious leaders……………….…….8 - Teachers………………………..…….9  Television…………………………….10 - Others (specify) …………………….11 |  |
| 403 | ***Check 303. If yes,***  When you went for antenatal visit, did you get any information related to cervical cancer from the health worker? | - No………..……0 - Yes…………….1 |  |

| 404 | In your opinion, who is at risk of cervical cancer?  A man, woman or any? |      | Men……………………...1  Women……………………2  Both men and women……..3 |  |
| --- | --- | --- | --- | --- |
|  |  |  | Others (specify) ………….10 |  |
| 405 | Have you ever known someone with cervical cancer? |    | Yes………………….……..1  No…………………………0 |  |
| 406 | Who can transmit the virus or germ that causes cervical cancer?  A man, woman or any? |      | Men…………………………1  Women………………………2  Both men and women………..3 |  |
|  |  |  | Don’t know………………......4 |  |
|  |  |  | Other (specify)……………….5 |  |
| 407 | Do you know how HPV/ germ that causes cervical cancer is transmitted/ contracted?  If yes,  In what ways is the virus or germ that causes cervical cancer transmitted? ***(Accept multiple responses or probe for more*)** |            | Unprotected sexual intercourse…………1  Pieced with sharp objects……………….2  Hereditary……………………………….3  Through body fluids like sweat…………3  Don’t know……………………………..4  Use of contraception……………………5 |  |
|  |  |  | Others (specify)……………….………...6 |  |
| 408 | Do you know the signs and symptoms of cervical cancer?  If yes,    What are the signs and symptoms of cervical cancer?  ***(Accept multiple responses or probe for more)*** |           | Pain in the genital during sexual intercourse1  Intermenstrual vaginal bleeding…………...2 Post menopausal vaginal bleeding………..3  Post-coital vaginal bleeding……………...4 Excessive vaginal discharge often with  offensive smell……………………………5 Lower abdominal pain……………………6 |  |
|  |  |  | Don’t know……………………………….7 |  |
|  |  |  | Others (specify)……………….………...8 |  |
| 409 | In your view, what behaviors increase the risk of getting the virus that causes cervical cancer?  **(Accept multiple responses)** |        | Smoking………………………………….1  Early onset of sexual activity……………2  Multiple male sexual partners…..………3 Infection with sexually transmitted  germ/virus (HPV)………………………4 |  |
|  |  |  | Multiparity……………………………..5 |  |
|  |  |  | Don’t know……………………………6 |  |
|  |  |  | Other (specify)…………………………7 |  |
| 410 | Mention different ways of preventing cervical cancer.  **(Accept multiple responses)** |      | Regular Screening………………………1  HPV vaccination………………………..2  Use of a condom………………………..3 |  |
|  |  |  | Abstinence from sex…………………….4 |  |
|  |  |  | Cervical cancer is curable in hospitals when  diagnosed early………………………….5 |  |
|  |  |  | Cervical cancer is preventable through genital exam by health providers (Pap smears)..6 |  |
|  |  |  | Faithfulness……………………………..7 |  |
|  |  |  | Don’t know………………………………8 |  |
|  |  |  | Others (specify)……………………….…9 |  |
| 411 | In your opinion, can cervical cancer be cured? |    | No………………..………..0 Yes……………….……….1 |  |

| 412 | Under what circumstances is a woman diagnosed with cervical cancer stand high chances of healing: when diagnosed late, when diagnosed early, at any time of diagnosis or you don’t know? |        | When diagnosed late……………….1  When diagnosed early……………..2  I don’t know……………………….3  Other (specify)…………………….4 |  |
| --- | --- | --- | --- | --- |
| 413 | Do you know how cervical cancer is treated?  If yes,    What are the different ways of treating cervical cancer  **Accept multiple responses** |          | By radiotherapy…………...…1  Removing uterus……………..2  Cervical cancer is curable in hospitals when diagnosed early……………....3  Seeing a traditional healer…...4  Taking herbs…………………5 |  |
|  |  |  | Don’t know………………......6 |  |
|  |  |  | Others (specify)……...………7 |  |
| 413a | **Total CC knowledge score**  ***To be filled after data collection*** |  |  |  |
| 414 | In your opinion how severe a disease is cervical cancer?  Is it very severe, somewhat severe, not severe at all or you don’t know? |        | Very severe…………1  Some what severe…...2  Not severe at all……...3  Don’t know…………..4 |  |

**SECTION 500; CERVICAL CANCER SCREENING**

| 501 | How can some body establish whether they have cervical cancer? |    | By going for cervical cancer screening…..1  Don’t know……………………………….2 |  |
| --- | --- | --- | --- | --- |
|  |  |  | Others (specify)………………………..….3 |  |
| 502 | Now I would like to talk about cervical cancer screening.  Have you ever heard of cervical cancer screening? |    | No……………..0 Yes……………..1 |  |
| 503 | How did you first learn about cervical cancer screening? |    | Newspapers and magazines..1  Radio……………………..2 |  |
|  |  |  | Billboards………………..3 |  |
|  |  |  | Family, friends, neighbours and colleagues.4 |  |
|  |  |  | Brochures………………...5 |  |
|  |  |  | Posters and other printed materials…6 |  |
|  |  |  | Health workers…………..7 |  |
|  |  |  | Religious leaders…………8 |  |
|  |  |  | Teachers…………………..9 |  |
|  |  |  | Television………………….10 |  |
|  |  |  | others (specify)…………….11 |  |
| 504 | In your view is it important for a woman to go for cervical cancer screening in her life time? |    | Yes………………………….1  No ………………………….0 |  |
| 504a | In your view how do you rate/ scale the importance of a woman attending cervical cancer screening in her life time: very important, important, not important, not important at all or you don’t know? |          | very important………………1 Important……………………2  Not important…………………3  Not important at all …………...4  I don’t know…………………..5 |  |

| 505 | In your view who is supposed to do Cervical cancer screening test: any person, a trained health worker, traditional healer or you don’t know? |        | Any person……………………1  A trained health worker………..2  Traditional healer………………3  I don’t know……………………4 |  |
| --- | --- | --- | --- | --- |
|  |  |  | Other (specify)………………….5 |  |
| 506 | On what part of the body is cervical cancer screening test done: any part of the body, female reproductive organ, or you don’t know? |        | Any part of the body……………1  Female reproductive organ……..2  I don’t know…………………….3  Other (specify)…………………..4 |  |
| 507 | In your view, what age category of women are recommended for cervical cancer screening test: any age, below 25 years, 2549 years, above 49 years or you don’t know? |        | Any age………………………….1  Below 25 years………………….2  25-49 years……………………..3  Above 49 years…………………..4 |  |
|  |  |  | I don’t know…………………….5 |  |
| **507a** | **Total knowledge score,**  ***To be filled after data collection.*** |  |  |  |

| NO. | **QUESTIONS AND FILTERS** | **CODING CATEGORIES** | | | **SKIP** | |
| --- | --- | --- | --- | --- | --- | --- |
| 508 | Do you know of any place where people can go for cervical cancer screening? | - No------------0 - Yes-------------1 | | |  | |
| 509 | Where can people go for cervical cancer screening: private, government or other health facility?  **Name of place……………** | - Public health facility-----------------1 - Private health facility----------------2 - Don’t know----------------------------3 - Other (specify)-------------------------4 | | |  | |
| 509b | ***check 509, If yes***  What is the walking distance **(in hours/kilometers**) from home to the screening place? *Probe and record hours separately from kilometers* | /          Hours / Kilometers | | |  | |
| 510 | Have you ever screened for cervical cancer | - No--------0 - Yes---------1 | | | **If no skip to 512** | |
| 511 | Where were you screened for cervical cancer: private, government or other health facility? | - Private facility-----------------1 - Government facility----------2 - Other(specify)-----------------3 | | |  | |
| 511a | What prompted /motivated you to go for screening? | - The desire to prevent illiness------------------1 - High perceived risk of infection---------------2 - Approval from partner--------------------------3 - After experiencing lower abdominal pain---4 - After experiencing vaginal discharge with   offensive foul smell----------------------------5   - After experiencing pain in the genital during   sexual intercourse------------------------------6   - After experiencing post coital vaginal bleeding--7 - Having some one in the family with cancer-8 - Others (specify)------------------------------9 | | |  | |
| 512 | In your opinion if given opportunity, would you accept cervical cancer screening?  If yes how would you rate or scale your acceptance? Definitely yes, probably yes, probably no, definitely no or You don’t know? | | - Definitely yes------------1 - Probably yes--------------2 - Probably no----------------3 - Definitely no---------------4 - Do not know----------------5 | |  | **Skip to 513** |
| 513 | Whom would you prefer to carry out cervical cancer screening? A male, female health worker or any? | |    | Male provider----------------------------1 Female provider--------------------------2 | |  |
|  |  | |  | Both male and female provider---------3 | |  |
| 514 | **Check 510. If no,**      Why have you not screened? | |        | Lack of time-------------------------------1  Fear of PAP test (including pain)-------2  Need more time with health provider---3  Test is too expensive----------------------4 | |  |
|  |  | |  | Test is too embarrassing-------------------5 | |  |
|  |  | |  | Too far to travel----------------------------6 | |  |
|  |  | |  | Lack of transportation---------------------7 | |  |
|  |  | |  | I am not at risk------------------------------8 | |  |
|  |  | |  | Long queues at serving points------------9 | |  |
|  |  | |  | Don’t know whether screening gadgets are sterilized | |  |
|  |  | |  | Other (specify)----------------------------11 | |  |

SECTION 600: HPV VACCINATION

| **No.** | **QUESTIONS AND FILTERS** | | | | | | | | | | **CODING CATEGORIES** | **SKIP** |
| --- | --- | --- | --- | --- | --- | --- | --- | --- | --- | --- | --- | --- |
| 601 | Now I would like to talk about vaccination done on girls of 10 to 12 years to prevent them from getting HPV(germ that causes cervical cancer)  Have you ever heard of HPV (germ that causes cervical cancer) vaccination? | | | | | | | | | | - Yes--------1 - No---------0 |  |
| 602 | How did you first learn about HPV Vaccination? | - Newspapers and magazines----------------------------1 - Radio------------------------------------------------------2 - Billboards-------------------------------------------------3 - Family, friends, neighbours and colleagues----------4 - Brochures------------------------------------------------5 - Posters and other printed materials-------------------6 - Health workers------------------------------------------7 - Religious leaders----------------------------------------8 - Teachers--------------------------------------------------9 - Television-----------------------------------------------10 - Others(specify)----------------------------------------11 | | | | | | | | | |  |
| 603 | To which category of girls is HPV Vaccine/ drug  administered: to any girl, young girls that have had sexual intercourse, young girls that have not had sexual intercourse or you don’t know? | - Any girl----------------------------------------------1 - Young girls that have had sexual intercourse---2 - Young girls that have not had sexual intercourse-3 - I don’t know----------------------------------------4 - Other (specify)-------------------------------------5 | | | | | | | | | |  |
| 603a | In your view, which age category of young girls is HPV vaccine administered to? | - Any age----------------------------1 - Below 9 years---------------------2 - 9-14 years--------------------------3 - Above 14 years---------------------4 - I don’t know-------------------------5 - Other (specify)----------------------6 | | | | | | | | | |  |
| 604a | In your view do you think HPV vaccination is beneficial to girls or your daughter? | - Yes------------------------------------1 - No-------------------------------------2 | | | | | | | | | |  |
| 604b | In your opinion how do you rate the benefits of HPV vaccination to girls or your daughter(s)? | - Very beneficial----------------------1 - Beneficial----------------------------2 - Not beneficial------------------------3 - Not beneficial at all-----------------4 - I don’t know--------------------------5 | | | | | | | | | |  |
| 604c | In your view, do you think it is very important to vaccinate young girls against HPV: | - Yes -----------------1 - No ------------------0 | | | | | | | | | |  |
| 604d | Why do you think HPV vaccination is important for young girls? | - It prevents young girls from contracting cervical   cancer in future----------------------------------------1   - Don’t know--------------------------------------------2 - Other (specify)----------------------------------------3 | | | | | | | | | |  |
| 605 | In your opinion who is supposed to administer HPV vaccine. | - Traditional healer--------------------------------------1 - Village Health Worker (VHTs)----------------------2 - Trained health worker---------------------------------3 - Any person--------------------------------------------4 - Other (specify)----------------------------------------5 | | | | | | | | | |  |
| 606 | Do you know any place where HPV Vaccination is done?  **If yes name of place-----------** | - Yes-----------------------------1 - No------------------------------0 | | | | | | | | | | If no, skip to 608 |
| 606a | What is the walking distance **(hours/ kilometers)** from home to the place where HPV vaccineation is done? *Probe and record hours separately from kilometers* |  |  |  |  | /  Hours / Kilometers |  |  |  |  | |  |
|  |  |  |  |  |  |  |  |  |  |  |  |  |
| 608 | How many shots/ doses is a girl supposed to get in order to complete the HPV Vaccine dosage: Once, twice or Other? | - Once---------------1 - Twice--------------2  I don’t know------3 - Other (specify)-----4 | | | | | | | | | |  |
| 609 | In you view what is the time interval between the shots/ doses: 6 months, 1 year or you don’t know? | - 6 months--------------1 - 1 year------------------2 - I don’t know----------3 - Other (specify)--------4 | | | | | | | | | |  |
| 610 | **Total HPV knowledge score,**  ***To be filled after data collection*** |  | | | | | | | | | |  |

I am going to ask you a number of questions. For each question answer yes, no or don’t know

|  | Questions | Yes……1 | No……2 | Don’t know..3 |
| --- | --- | --- | --- | --- |
| 611 | Would your husband/partner accept/ support HPV vaccination for your daughter(s)? |  |  |  |
| 612 | In your opinion, are you able to access a trained health worker for the vaccination of your daughter(s)? |  |  |  |

| 613 | Now I would like to talk about cervical cancer risk factors.    Do you smoke? |    | Yes---------------1  No----------------0 |  |
| --- | --- | --- | --- | --- |
| 614 | Do you drink alcohol? |  | Yes----------------1 |  |
|  |  |  | No-----------------0 |  |

Check 305, if yes,

**Now, I would like to record the names and other details related to HPV vaccination for the…. (number (check 305)) girls starting from those of 10 years to those aged 17 years.**

Record names of all girls in the household aged 10-17 years in 615

| **614** | **615** | **616** | | |  | **617** | **617a** | **618** | **619** | **620** | **621** | **621a** | **622** | **623** |  |
| --- | --- | --- | --- | --- | --- | --- | --- | --- | --- | --- | --- | --- | --- | --- | --- |
|  |  |  | | |  |  |  |  | ***Check 618***  ***If yes,*** |  |  | Check  621 if  Once, | ***Check 620***  ***If yes*** | ***Check 620 if yes*** |  |
| Mother’s    Code/ id | Child’s code/ id    **Name** | Age in years | | |  | Parity (Birth order) | …(name)  Relation-  Ship to the respondent **A=Biological Child..1**  **B=Other Relationship**  **(specify).2** | Is…(name  ) currently attending school? **A=Yes..1**  **B=No….0** | What  is the type  of school  **A=Governm**  **ent..1**  **B=Private..2**  **C=Others**  **(specify)..3** | Was (name) vaccinated  against  HPV –To **check cards if available**  **A=No--0 B=Yes--1**  **C=Don’t Know-2** | How many ti  mes?  **To check**  **cards A=Once B=Twice** | Time  in  months since Last dose | Where was she vaccinated from? **A=School..1**  **B=Home…2**  **C=Health facility….3** | Who decided when  ...(name) was vaccinated? **A=wife**  **B=spouse**  **C=Both D=None**  **E=Other (specify)** |  |
|  |  |  | | |  |  |  |  |  | **If no skip to 625** |  |  |  |  |  |
|  | 01 |  |  |  |  |  |  |  |  |  |  |  |  |  |  |
|  |  |  |  | |  |  |  |  |  |  |  |  |  |  |  |
|  | 02 |  |  |  |  |  |  |  |  |  |  |  |  |  |  |
|  |  |  |  | |  |  |  |  |  |  |  |  |  |  |  |
|  | 03 |  |  |  |  |  |  |  |  |  |  |  |  |  |  |
|  |  |  |  | |  |  |  |  |  |  |  |  |  |  |  |
|  | 04 |  |  |  |  |  |  |  |  |  |  |  |  |  |  |
|  |  |  |  | |  |  |  |  |  |  |  |  |  |  |  |

**…………………………………………..Ta**

**ble continued**

| \| **615** \| **624** \| **625** \| **626** \| **627** \| **628** \| **629** \| \| --- \| --- \| --- \| --- \| --- \| --- \| --- \| \|  \| ***Check 620 if yes*** \| **Check 620**  **If no,** \| **Check 620 if no,** \| **Check**  **620 if no,** \| **Check**  **620 if no,** \| **Check**  **620 if no,** \| \| Child’s code/ id          **Name** \| What form of support did your spouse offer for …(name) to be vaccinated? **A=Counselling..1**  **B=Transp-t…..2 C=Others**  **(specify)…….3** \| what were the reasons for not being vaccinated? **Check right corner for codes** \| Would you allow (name) vaccinated in the next six months if given opportunity?  **A=Yes…1**  **B=No….0**  **C=Don’t know** \| Did your husband object the  vaccination of ..(name)  **A=yes.1**  **B=No..0** \| How  did he object the  vaccinatio n of ..  (name) \| For (name)  what were the reasons for objectttingthe vaccineation by the husband? \| \|  \|  \|  \| ***Check 620 if yes skip to 701*** \| **If no, skip to 701** \|  \|  \| \| **01** \|  \|  \|  \|  \|  \|  \| \| **02** \|  \|  \|  \|  \|  \|  \| \| **03** \|  \|  \|  \|  \|  \|  \| \| **04** \|  \|  \|  \|  \|  \|  \| | |  |
| --- | --- | --- | --- | --- | --- | --- | --- | --- | --- | --- | --- | --- | --- | --- | --- | --- | --- | --- | --- | --- | --- | --- | --- | --- | --- | --- | --- | --- | --- | --- | --- | --- | --- | --- | --- | --- | --- | --- | --- | --- | --- | --- | --- | --- | --- | --- | --- | --- | --- | --- | --- | --- | --- | --- | --- | --- | --- | --- |
| **CODES FOR Q 625 $ 629**  **A**=Lack of knowledge about HPV----1  **B**=She is too young to be vaccinated--2  **C=**She is not at risk----------------------3  **D=**Not certain of the safety of the vaccine-4  **E=**My child my become infertile in future-5 **F=**Fear of increase in daughter’s risky sexual behaviors-------------------------------6 **G=**Lack of access to the vaccine-----------7 **H=**I don’t know whom to hold responsible of the vaccine consequences----------------8 **I=**Others (specify)---------------------------9  **CODES FOR Q 628**  **A=**Stopped the child from attending school that day----------------------------------1 **B=**He sent away health workers from  home---------------------------------------2 **C=**Assigned the child alternative assignments at the time of vaccination-3 **D=**Threatened the child that the exercise had side effects---------------------------4  **E=**Others (Specify)----------------------5 | |  |

**SECTION 700; SPOUSE/ PARTNER’S FACTORS**

| **NO.** | ***QUESTIONS AND FILTERS*** | **CODING CATEGORIES** | | | | | **SKIP** |
| --- | --- | --- | --- | --- | --- | --- | --- |
| 701 | ***Check -104b- If currently married***  Now I would like to talk about issues related to your husband/ spouse.  How old is your husband? |  | | | | | ***Check 104a If not in any union skip***  ***to closing remarks*** |
|  |  |  |  |  |  | Years |  |
| 702 | `What is the education level of your husband? | - No education---0 - Primary-----------1 - Secondary--------2 - Tertiary-----------3 - University--------4 | | | | |  |
| 703 | Do you always seek for permission before seeking for medical care? | - Yes-----------------1 - No------------------0 | | | | |  |
| 704 | Under what circumstances do you seek for permission before seeking medical care? | - In all circumstances-----------1 - When ill-------------------------2 - In case of distant referrals-----3 - Others (specify)-----------------4 | | | | |  |
| 704a | Who usually makes decisions about health care for yourself: you, your (husband/ partner), or you and your (husband/ partner) jointly or someone else? | - Respondent-----------------------------------1 - Husband/ partner-----------------------------2 - Respondent and husband/ partner jointly--3 - Someone else----------------------------------4 - Other (specify)--------------------------------5 | | | | |  |

PARTNERS’ ROLE IN CERVICAL CANCER SCREENING

| 705 | Have you ever sought permission from your husband to go for cervical cancer screening? |    | No--------------0  Yes------------1 | **If no, skip to closing remarks** |
| --- | --- | --- | --- | --- |
| 706 | When you sought permission to go for screening what was his response? |    | Rejected/ declined-------0  Accepted -----------------1 | ***If 0, Skip to 708*** |
| 707 | If he accepted what form of support did he offer? |    | Financial------------------1 Money for transport-----2 |  |
|  |  |  | Encouragement----------3 |  |
|  |  |  | Information---------------4 |  |
|  |  |  | Other (specify)-----------5 |  |
| 708 | **Check 706 if 0,**    What were the reasons for declining the suggestion?      **Accept multiple responses** |            | Lack of time---------------------------1  Fear of PAP test (including pain)--2  Test is too expensive-----------------3  Test is too embarrassing------------4  Too far to travel----------------------5  Lack of transportation---------------6 |  |
|  |  |  | Others (specify) -----------------------9 |  |
| 709 | What did you do when he refused you to go for screening? |    | I went against his will-----------------------1 I waited until he gave me the money------2 |  |
|  |  |  | I am waiting for him to get for me money-3 |  |
|  |  |  | I am waiting for him to permit me----------4 |  |
|  |  |  | Others (specify)-------------------------------4 |  |

***Thank you very much for participating in our survey.***
