# Supplementary material for: Predictors of cervical cancer screening uptake in two districts of Central Uganda
Source: PLoS One. 2020 Dec 3;15(12):e0243281. doi: 10.1371/journal.pone.0243281 (PMC7714132; doi:10.1371/journal.pone.0243281)
Supplement: Attachment — Submitted filename: swanson_reviewnotes_isabirye.docx [file pone.0243281.s004.docx]

Review notes

Abstract:

The Background section (lines 8-10), should state the problem (which is 2 fold: high burden cervical cancer, low rates of screening) and the objectives of this study, which I also see as two-fold: report prevalence of screening in these districts as well as investigate associations between various socio-demographic predictors and screening.

Methods: line 13 – pick either “univariate” or “bivariate” – they are the same.

Results: Line 15: age – is this relatively older or younger age? Line 15: source of *what* information? *Health* information? Line 15/16: the “knowledge” variable needs to be better defined throughout – I would re-define it here as “knowledge of importance of screening to prevent cervical cancer” or as an “understanding of the role of screening in cervical cancer prevention.” Similarly, the “logistics” variable needs to be better defined and I would re-define here as “knowing where to go for screening,” as you do in line 20. The odds ratios (lines 16-21) should be worked in with the prior sentence stating significant predictors. Please add “95%” prior to CI in lines 16-21. Also, please make clear if this is bivariate or multivariate analysis.

Conclusions: The conclusion needs to be re-written. Lines 23-24 only include some of the significant findings. Lines 25-30 go beyond the scope of the data. The first sentence is the overall finding and is good (line 22). I would limit the conclusion to 2-3 sentences in total. For the concluding sentence, I would offer something like: “relatively greater access to resources and knowledge, both disease-related and logistical, predicted a higher prevalence of screening” and then OK to have a final sentence with data-based recommendations for future. The last sentence could be something like: “efforts to increase uptake of screening must address disparities in access to resources and knowledge.”

Background:

In general, the first 2 paragraphs should be combined into 1 (or 2, but re-organized) paragraph(s) painting the picture of cervical cancer in LMICs and Uganda specifically, then you should have a paragraph about what is known about screening, then a description of this particular study’s goals.

Lines 33/34: make 1 sentence about ½ million new cases and 1/3 million deaths annually and cite Bray 2018. Take out the global age-standardized incidence rate as you discuss later (line 50) and it makes more sense there.

Lines 35/37: be careful about saying “disproportionately” as 85% of world’s population lives in LMICs, according to the World Bank, so 70% is not technically “disproportionate.” I think the 70% number is not accurate – The GLOBOCAN estimates more like 84% of new cases and 87% of the deaths occur in LMICs. You cite Torre 2012 as citation #2, but your citation for #1, the Bray GLOBOCAN estimates should replace the 2012 estimates. Take out citation 2 and update.

Line 37: Do you mean “sub”-Saharan Africa?

Lines 38-40: Take out the India statistic and it’s confusing to also hear about breast cancer – I would just focus on the incidence and mortality rates, for which Southern and Eastern African regions are the highest in the world. Be careful: your references #3 and #4 are the same and I’m not sure are complete.

Instead of 41-45: I would end this paragraph by saying something like: “while widespread cytology-based screening in high-income countries has resulted in decreased incidence and mortality from cervical cancer, LMICs, with poor uptake of screening, have not seen similar improvements and, in some cases, incidence and mortality actually continue to rise.” If you want to include the WHO recommendations, I would instead use the WHO guidelines for screening and treatment of precancerous lesions for cervical cancer prevention, 2013. Your statement in line 42 about screening (do you mean once per lifetime screening recommended for 25-49yos?) is an over-simplification of the recommendations. Not sure the SDGs add and reference #6 seems to be incomplete.

Lines 47-64: this paragraph is all Uganda-specific. It’s fine to follow the first general paragraph about high prevalence cervical cancer and low prevalence screening in LMICs, but this paragraph needs to be shorter and more focused:

- Lines 47-49: summarize that Cervical cancer is most common incident and mortal cancer among women in Uganda
- Lines 49-51: one sentence with age-standardized incidence and mortality rates in UG compared to global OK to underscore the magnitude of the problem. Be careful of your citation, I would use the Bruni HPV Uganda-specific report for the citation – make sure the citation is complete (For example, I have cited as: Bruni L B-RL, Albero G, Aldea M, Serrano B, Valencia S, Brotons M, Mena M, Cosano R, Muñoz J, Bosch FX, de Sanjosé S, Castellsagué X. Human Papillomavirus and Related Diseases in Uganda. Summary Report 2016- 02-26: ICO Information Centre on HPV and Cancer (HPV Information Centre), 2016.)
- Lines 52-56 can essentially be cut as long as you have the summary statement about cervical cancer as the most common incident and mortal cancer among women in uganda.
- Line 56-57: cut this sentence about 8/10 women at UCI have cervix cancer. Reference 9: seems incomplete the way it is cited and probably OK to just cite the GLOBOCAN 2018 instead of the Uganda Fact Sheet.
- Lines 57-60: I’m not sure what these lines add to the intro. This is not a paper about cancer, it’s a paper about screening, so I think sufficient to make a grim summary statement about cervical cancer earlier on. I would cut these sentences and references 10-12 (also, these references seem incomplete).
- Lines 60-64: this is a good way to end this paragraph, to bring it back to Uganda. Lines 60-63 should be one sentence about the MOH goal to screen 80% - be careful, your citation for #13 is incomplete: change to “MOH. Strategic Plan for Cervical Cancer Prevention and Control in Uganda, 2010-2014. Kampala, Uganda: Ministry of Health; 2010.” Line 63-64 – what study are you citing here? Citation 14 is incomplete? It might be stronger to start the next paragraph with the best estimate of screening prevalence in Uganda.

Lines 66-85: This paragraph is too long and is disorganized. It is a good idea to use this paragraph to discuss what it is we know about screening and why we need to know more in order to improve screening programs. Some of what is written in this paragraph should be removed and saved for the discussion where you will compare this study’s findings to that of other studies.

- The paragraph could start with your statement about how research on determinants play a role in designing effective interventions (lines 69-70), but its not clear to me that your references 17-21 (careful, most of these references are incomplete and be consistent with either numbering) actually justify this claim – are you suggesting that these studies used some sort of implementation science approach to designing their interventions?
- Lines 72-73 say another general statement about how cervical cancer is preventable if screening is implemented – your citations 22 and 23 don’t really justify this statement which is generally accepted to be true, and the citations are incomplete. This general statement does not belong at this point in the introduction.
- Lines 74-76 and 66-68 go together and given an overview of what is known about cervical cancer screening – be careful of your references, some are incomplete. I would stick to references about Eastern Africa.
- Lines 76-81 focus on urban vs rural – the first sentence is incomplete and unnecessary. The next 2 sentences are too long are not focused (Lines 77-81) – are you just trying to say that living in a rural area is predictive of not screening?
- Lines 81-85 talk about “strengths” of the current study – this goes in discussion, not background. Instead, end the paragraph with a statement about the objective of the current study. Something like, “the objective of the current study was to report the prevalence of screening in Central Uganda and to examine for associations between predictors and successful screening in order to inform the design of future screening programs…”

In general, this is a paper about screening and I am surprised that I don’t find anything in the introduction about VIA versus HPV vs cytology for methods screening. Generally speaking, there is too much about cervical cancer and not enough about screening.

Methods:

The first section should include not only a description of study setting, but also study population – you can include the age-range here… other inclusion/exclusion? Language?

Line 95: reference 34 is incomplete.

Lines 95-97: you claim there is no precedent in the literature, but in line 110, you cite studies which likely report on a prevalence of screening. There are estimates out there – if you think they are underestimates (50% is high), you need to explain why you think prior studies would be underestimate and so you’ve gone with 50%.

Lines 97-98, I think you have overestimated your sample size. You could say something like, “To calculate our sample size, we assumed that if 50% of women in Central Uganda had previously screened for cervical cancer, a total of 402 women would allow us to experimentally determine this proportion within 5% (confidence level 95%) using a binomial “exact” calculation (Hulley SB, Cummings SR, Browner WS, Grady D, Newman TB. Designing clinical research : an epidemiologic approach. 4th ed. Philadelphia, PA: Lippincott Williams & Wilkins; 2013. Appendix 6E, page 81). <https://www.sample-size.net/sample-size-conf-interval-proportion/>

Lines 98-100: this is not a “cluster randomized study,” rather, you used “cluster sampling” to select a simple random sample of clusters. This is very different and needs to be clarified.

Lines 99-101: Why 70% from Wakiso and 30% from Nakasongola? Explain and provide justification.

Line 101: “the target population…” – this belongs in “inclusion criteria.” Reference 35 does not make sense here. If you are justifying the ages in your inclusion criteria, I would cite Ugandan MOH recommendations for screening a particular age range (they recommend 25-49).

Lines 101-103 should be removed here – this is for inclusion criteria. For sampling procedure, describe the cluster sampling technique.

Lines 109-110: Were any of the questionnaires validated? Doesn’t make sense to say that the questionnaire was adapted from “findings” from other studies – sounds like it was modified from questionnaires used in other studies.

Lines 119-124: should be a separate paragraph.

Lines 124-127: the description of the research assistants and their training could be a separate paragraph.

Lines 127-128: talk more about the pre-test/pilot phase – were questions adapted? Community participation is an important part of study design and this could be highlighted here.

Lines 128-129: remove the sentence “the questionnaires were administered by 5…” – this is redundant.

Lines 129-131: this is a good way to summarize, but also, were data electronically captured? Sent to a secure server?

Line 134: a cervical examination is not screening – do you mean VIA? This has to be better defined.

Line 135-136: screening does not test for cervical cancer, but also pre-cancer/dysplasia…

Lines 133-161: this paragraph is way too long and needs to be split up. You don’t need to include so much information about basic demographic variables, just need to say that demographic info including age, religion, place residcen, ethnicity, marital characteristics, parity; as well as health characteristics like use fam planning, recent visit to health center. Lines 147-148: this is the first I’m hearing about this “autonomy” variable – I did not see in abstract, if not used in analysis, don’t need to describe. The description of the “knowledge” variable 149-157 is too long and should be truncated/summarized. Line 157-158 – the justification doesn’t need to be there and the reference 39 is incomplete and doesn’t make sense. Lines 158-161 – description wealth index is too long and also was presented in abstract as dichotomous (rich/poor).

There is description of the “source of information” variable – as that is not an obvious variable, please explain – source of *what* information, exactly?

Line 164: I don’t understand how Epidata was used, clarify.

Lines 166-171 need to be re-written. I image you used chi-square tests to test for association between outcome (being screened ever) and categorical explatory variables. And I imaging that for continuous explanatory rpedictors, you used t-tests to compare sample means by outcome. I imagine you used logistic regression to explore association demographic and health-related predictors with outcome, adjusting to limit bias from confounding. Discuss how you build the multivariate model and whether sensitivity analyses were performed.

Line 168: you sat that knowledge is a dependent variable, yet you treat it an an independent predictor and your dependent variable is your outcome variable, screening. Fix this.

Results

The results section needs to be rewritten. The section needs to lead with the proportion who had ever been screened (your main outcome).

Tables 1 and 2 should be combined into one table, I would suggest a table that looks like this:

| Characteristic | Total (%) | History prior screening (%) | Never been screened (%) | OR unadjusted prior screening (95% CI) | OR adjusted prior screening (95% CI) |
| --- | --- | --- | --- | --- | --- |
| Total (N, %) | 845 | XXX (21%) | XXX (79%) |  |  |
| Age |  |  |  |  |  |
| <=29 | 315 (37%) | 13 | 87 | 1.0 | 1.0 |
| 30-39 | 364 (43%) | 23 | 77 | 1.4 (0.83-2.19) | x.x (a-b) |
| 40-49 | 166 (20%) | 30 | 70 | 1.9 (1.05-3.55) | x.x (a-b) |

Some specific feedback:

Line 192: remove sentence “results in table one…”

Lines 192-200 – the order of the variables needs to be re-organized. For example – Catholics are mentioned in 196 and then protestants are mentioned in line 199. You are describing the population in this paragraph, which is a fine was to start.

Line 202-203 : remove this first sentence. Lines 203- 212: this needs to be made into one paragraph and re-organized. Also, are you discussing bivariate or multivariate analysis? What is going on with the “knowledge” variable? In the Methods section, you discuss a composite score variable, but here there are two knowledge variables – please be consistent.

Lines 223-225 – remove this sentence.

Lines 227-235 – need to be combined and re-written with lines 192-203. This is all the same information.

Some other thoughts about the variables (in table one):

- Age – do you need three categories? Often easier to understand as a dichotomous variable
- Religion – consider combining as Christian versus Muslim
- Study site: note which is urban/which is rural
- Education: consider a dichotomous variable – completed primary (at least some secondary) versus not completed (some primary)
- Occupation: consider professional versus other (“proffessional" is spelled wrong)
- Marital status: consider married vs single/widow/separated
- Ethnicity: consider combining for fewer categories
- Age at first marriage: the “single” category should not be in there.
- Knowledge: this is different than described in Methods and is different table one to table two. Make consistent
- Distance to screening facility: “don’t know where to go” does NOT belong in there – that is a different variable… is that different than “awareness of screening services?”

Discussion

Overall, this is the best-written section of the paper, but it still needs significant work.

Lines 240-246: good to start with your overall finding about screening prevalence. I would take out the first sentence. I don’t think it’s necessarily useful to include stats from Nepal, I would stick to the region.

Lines 248-252: this is about knowing where to go – the logistics variable. I would remove the words “the possible explanation for this finding is that” and just start the sentence with “Increased awareness of …”

Lines 254-259 are about the age variable. This paragraph is OK.

Lines 260-269: this paragraph is about he “source of information” variable. As I’ve mentioned before, this variable needs to be better defined. I would remove the sentence lines 263-265 “this observation might be due to..” I would also remove the sentence lines 267-269 “Secondly it…) I would add a sentence positing that health workers may be essential in health messaging.

Lines 271-274, this paragraph is about the wealth variable – I would re-write the first sentence – take out “corrobortating the results…” part. Overall the paragraph is ok.

Lines 276-281, the “knowledge” variable is problematic and I have brought that up before. I would take out he sentence 278-279 “understanding the impotance…” – this is speculation. Your last sentence is an important point that prior screening is likely related to increased knowledge.

Lines 284-286: Study limitations: can’t assess causality and generalizability. What about sources of bias?

You have two nearly identical paragraphs: recommendations and conclusions – this should just be there once, likely as “conclusions”

Lines 289-295: take out “substantial effect” line 290 and change to something like “associated” – you cannot imply causation. You then focus on the source of information variable and suggest that antenatal providers should discuss screening – this is a really important point and should be developed! Why not screen in pregnancy? What proportion of women interact with providers during pregnancy (at least one anenatal visit)? This also brings in the knowledge variable – be explicit about how these are related. You then focus on the wealth index variable and suggest universal healthcare – what are some challenges there? Does the government have the funding? What would make cervical cancer screening cost effective?

References (lines 317-423) – nearly all the references are incomplete – please be careful and re-do the references.
