# Supplementary material for: Predictors of cervical cancer screening uptake in two districts of Central Uganda
Source: PLoS One. 2020 Dec 3;15(12):e0243281. doi: 10.1371/journal.pone.0243281 (PMC7714132; doi:10.1371/journal.pone.0243281)
Supplement: Attachment — Submitted filename: Response letterPLOSOne.docx [file pone.0243281.s005.docx]

**Response letter**

| **Date** | 12 July 2020 |
| --- | --- |
| **To:** | PLOS ONE <plosone@plos.org |
| **From:** | "Alone Isabirye" aloneisab@gmail.com |
| **Subject:** | Response to review comments of our manuscript submitted to PLOS ONE (PONE-D-20-02899) |

PONE-D-20-02899
Predictors of Cervical cancer screening uptake in Central Uganda
Alone Isabirye, Martin Mbonye, Betty Kwagala
PLOS ONE

Dear Editor,

Thank you for your reply regarding our manuscript "Predictors of Cervical cancer screening uptake in Central Uganda" (PONE-D-20-02899). We are grateful for your and the reviewers’ comments. We have revised and modified the manuscripts according to your and referees’ critiques. As a consequence, we provide a revised manuscript with your and reviewers’ suggestions integrated therein:

**Response to academic editor’s comments**

1. The manuscript was prepared following PLOS ONE’s style requirements, including those of file naming
2. Validation of the questionnaire was described.

- A copy of the questionnaire in both the original language and English as supporting information were uploaded

- The type of informed consent obtained was specified in the methodology section and online submission information was updated.

3. My ORCID iD was validated in Editorial Manager
4. Data set was uploaded as supporting information.

**Response to reviewer 1’s comments**

**Abstract**

**Background**

The problem, objective, prevalence of screening, as well as investigate associations were included.

**Methods**: We used bivariate

**Result**: Age was not reflected in the abstract because it was not significant when we reanalyzed data.

Source of information was clarified

Knowledge variable/ score was well defined

The logistic variable was included among the knowledge scores

Prior sentence stating significant predictors was included

We add “95%” prior to CI

The results are clearly stated as multivariable results

**Conclusion**: The conclusion was rewritten as suggested

All significant findings were included

Recommendation that were outside the study scope were excluded

Conclusion is summarized in three sentences

The concluding statement was adopted

**Background**

This section was organized into three paragraphs with a description of the picture of cervical cancer in Low and Middle Income Countries (LMICs), what is known about screening, and a description of the study goals.

Lines 33/34 were rephrased and summarized. Appropriate references were cited

Lines 35/37: The word disproportionate was eliminated and relevant facts were presented backed by appropriate citations.

Line 37: The right word “Sub-Saharan” was used

Lines 38-40: India statistic was removed, Section about breast cancer was removed, references were harmonized and checked for completeness. Much focus was put on mortality and incidence especially in Eastern Africa.

Instead of 41-45: We used a suitable statement as recommended. Appropriate citations were included and the incomplete references were fixed.

Lines 47-64: The paragraph was summarized

Lines 47-49: A summarized statement was presented

Lines 49-51: Appropriate references were cited

Lines 52-56: The statement was cut short

Lines 56-57: The sentence was summarized and appropriate reference cited

Lines 57-60: These statements were dropped

Lines 60-64: The reference was fixed

Lines 66-85: The paragraph was summarized. Only important information for the section was retained and other details were used to discuss the findings.

Lines 69-70: Clarification was provided about the nature of studies related to the role of evidence of predictors.

Lines 72-73: The statement was transferred to appropriate section and relevant literature cited.

Lines 74-76 and 66-68: Information from these sections was merged to create coherence. We mainly considered studies from Eastern Africa

Lines 76-81: The unnecessary statement was eliminated.

Lines 77-81: The sentences were summarized and refocused

Lines 81-85: The objective of the study concluded this section

**General observation:** The section was presented with a balanced discussion about cervical cancer and cervical cancer screening.

**Methods**

The first subsection of the methods sections included the study setting, the study population, and inclusion/ exclusion criteria.

Line 95: Reference checked and fixed

Lines 95-97: The authors’ aim of using 50% prevalence was to obtain maximum possible sample size which provided more precise estimates.

Lines 97-98: The authors think that the sample size presented is not overestimated because it was rigorously derived with important assumptions fully catered for by the approach employed. Such assumptions included a consideration of the study design, nonresponse rate, and other key assumption that are not accommodated by other formulae.

Lines 98-100: The statement was corrected to “cluster sampling”

Lines 99-101: The 70% and 30% from Wakiso and Nakasongola respectively were allocated because Wakiso had the biggest number of eligible participants. It was in the authors’ interest to allocate the sample size using proportional fraction but the population for Wakiso district was extremely big to the extent that it would leave unreasonable subsample for Nakasongola district. Therefore, the authors decided to select 40% more women from Wakiso district as a way of accommodating for the imbalance in the distribution of the eligible population between the two districts.

Line 101: Target population was transferred to an appropriate position and an appropriate reference was cited.

Lines 101-103: These statements were shifted to introductory part of the section and the cluster sampling technique was explained.

Lines 109-110: An explanation of validation of questionnaires was provided.

Lines 119-124: Were presented in a separate paragraph

Lines 124-127: The description of the Research Assistants and their training were presented in a different paragraph.

Lines 127-128: Pretesting explained in details. We indicated that questions were adapted. Community participation is reflected especially our research assistants and the involvement of the local leaders.

Lines 128-129: The redundant statement was removed

Lines 129-131: `Data was collected using questionnaires, captured/ entered into epidata software, and transferred to stata software for analysis. Epidata is a software that was designed specifically for entering data that is already existing.

Line 134: The right word “VIA” was used

Lines 135-136: The paragraph was summarized

Lines 147-148: The “autonomy variable” was removed

Lines 149-157: The description for the “knowledge variable” was summarized.

Lines 157-158: The justification was removed

Lines 158-161: The wealth index was summarized. In The abstract, we only presented two categories that were statistically significant even when the variable had three categories.

The right statement “Source of information of screening” was used

Line 164: Epidata is the software we used to capture/ enter our data that was collected with the questionnaires.

Lines 166-171: We analyzed data at bivariate level first for both categorical and continuous variables using chi square tests. Continuous variables like age and parity were transformed into categorical variables. After chi square tests all variables that were significant at bivariate analysis were simultaneously analyzed using a multivariable logistic regression model. We performed a multicollinearity test.

Line 168: Knowledge variable was presented as an independent variable throughout.

**Results**

The section was rewritten. The section lead with proportion of women who had ever been screened.

Table 1 and 2: We appreciate that using the unadjusted odds ratios would reduce the number of tables to one. But the authors suggest that since we had already used the chi square tests to serve the function the unadjusted odds ratios would serve, they propose that the number of tables be left as they were with slight adjustment in their formatting.

Line 192: Sentence “results in Table one” removed

Lines 192-200: The order of variables were reorganized

Lines 202-203: The sentence was removed

Lines 203-212: This was reorganized and presented as one paragraph. We are discussing bivariate results. The knowledge variable was reorganized.

Lines 223-225: The sentence was removed

Lines 227-235: The sections were rewritten but were not combined since PLOSONE requires that tables should appear shortly after the paragraphs where they are cited.

Some other thoughts about the variables (in Table one):
- Age – The variable was dichotomized
- Religion – The variable was dichotomized
- Study site: Study sites were noted as either rural or urban
- Education: The variable was dichotomized
- Occupation: The variable was dichotomized
- Marital status: The variable was dichotomized
- Ethnicity: Some categories were combined
- Age at first marriage: the “single” category was recoded.
- Knowledge: The description was harmonized with what was presented in the tables
- Distance to screening facility: the “don’t know where to go” category was recoded

**Discussion**
The comment was appreciated

Lines 240-246: The first sentence was removed and priority was given to references from the region.

Lines 248-252: These were scored as knowledge variables after reanalyzing data

Lines 254-259: Thank you for the comment. But on reanalysis with fewer categories, age became insignificant.

Lines 260-269: The variable was well defined. All unnecessary phrases were removed.

Lines 276-281: The knowledge variable was explained. All unnecessary statements were eliminated.

Lines 284-286: Sources of bias were highlighted

The two identical paragraphs were dissolved into one (conclusion)

Lines 289-295: “Substantial effect” removed. This section was reorganized and appropriate explanations were presented.

References (Lines 317-423) were revisited and corrected.

**Response to reviewer 2’s comments**

**Response to general comments**

- The authors revised the background with more information about cervical cancer screening. Available methods of screening were presented and problems of lack of knowledge of predictors of cervical cancer screening were presented.
- Table one was presented in an informative manner as suggested
- The title was amended because the two districts are not a representation of the 27 districts

**Specific comments**

- Line 1: The title was changed to “Predictors of cervical cancer screening uptake in two districts of central Uganda” since the two districts are not a representation of the 27 districts
- Line 13: The type of statistic and the outcome it measured are stated.
- Lines 41-45: Statement eliminated
- Line 51: “25” was written as “25.0” and “Global” as “global”.
- Lines 81-82: The overall aim of the study was fully stated
- Lines 83: Statement eliminated
- Lines 90-92: Some adjustments were made in the title.
- Lines 98-99: The statement “cluster random study” was replaced with random sampling of clusters. Villages/ wards were described as grouping clusters. We selected a rural and an urban district from Central Uganda.
- Lines 99-101: The 70% and 30% from Wakiso and Nakasongola respectively were allocated because Wakiso had the biggest number of eligible participants. It was in the authors’ interest to allocate the sample size using proportional fraction but the population for Wakiso district was huge to the extent that it could leave unreasonable sub sample for Nakasongola district. Therefore, the authors decided to select 40% more women from Wakiso district to accommodate for the imbalance in the distribution of the eligible study participants in the two districts.
- Line 102: It is Uganda’s ministry of health and WHO that recognize them as high risk age group.
- Lines 104-105: 24 villages from Wakiso of the 34 villages considered for the study is 70% and 10 villages from Nakasongola represent 30% of the studied clusters.
- Line 128: The pretesting was explained
- Line 128-130: The numbers were presented logically
- Line 178: We sought written informed consent and corrections regarding this was made
- Line 193: Though the sample size was not assigned using a formula it was intended to reflect the imbalance in the distribution of the eligible population in the two districts. Nevertheless, the overall sample size was derived using a rigorous process.
- Lines 192-200: The “%” was placed wherever it was missing.
- Line 218 (Table 1): The table was improved as suggested.
- Lines 225: The criteria for selecting variables for the multivariate logistic regression was described.
- Lines 244-246: References for the hospital removed and explanation adjusted.
- Lines 255-256: What other similar studies established was emphasized
- Line 268: This variable was insignificant on reanalysis.
- Lines 289-29: Statement deleted
- Line 293-295: The recommendation was removed
- Lines 297-298: Results were removed from the conclusion section
- Lines 300-305: This was merged with earlier section into conclusion
- Lines 350-351, 365, 398, and 413: All references were checked and fixed.

We hope that our modifications render our manuscript in its current form suitable for publication in PLOSONE

Yours sincerely,

Isabirye Alone

aloneisab@gmail.com

**On behalf of the authors**
