# Supplementary material for: Predictors of cervical cancer screening uptake in two districts of Central Uganda
Source: PLoS One. 2020 Dec 3;15(12):e0243281. doi: 10.1371/journal.pone.0243281 (PMC7714132; doi:10.1371/journal.pone.0243281)
Supplement: Attachment — Submitted filename: swanson_reviewnotes1025_isabirye.docx [file pone.0243281.s006.docx]

Reviewer Notes

Predictors of cervical cancer screening uptake in two districts of Central Uganda

Overall, this is a much stronger version. Thank you for working to revise the manuscript. The following are minor suggestions for revision.

Abstract:

- I think you can remove lines 13-14
- Line 16, after “targeted interventions”, I would add “to improve screening”
- Line 26, as you are shifting to conclusion, I would add the word “Overall,” cervical cancer screening low…
- Lines 29 and 30: remove “the above findings suggest that” and start this sentence with “Efforts to increase uptake…”

Background: In general, this is much improved with the emphasis on screening, rather than cancer, but I still think the section could be shortened.

- I would take out some of the cancer-specific stats from paragraph one and transition quicker to the problem of inadequate screening. Consider splitting into two paragraphs.
- Line 57: need to write-out “Visual inspection with acetic acid” before VIA
- Line 64: You already defined LMICs
- Lines 63-67: consider removing these lines and starting with “the success of screening depends on uptake…” – also depends on follow-through the cascade to treatment. Vaccination is beyond the scope of your discussion. If you are going to mention vaccination, I would say something to the effect of “while comprehensive vaccination is cost-effective and lifesaving, incidence of cervical cancer not expected to drop for at least 2 decades after widespread vaccination uptake (Jit M, Brisson M, Portnoy A, Hutubessy R. Cost-effectiveness of female human papillomavirus vaccination in 179 countries: a PRIME modelling study. The Lancet Global health. 2014) and in the meantime cervical dysplasia and early cancers will remain common and treatable”
- Lines 72-79: Be careful, comparing various screening methods is beyond the scope of this paper – the debate between HPV testing and VIA is nuanced and there are strong feelings on both sides.

Methods:

- Line 181-182: in forward-stepwise model building, usually cast a wider net and include predictors with p value more like 0.2 or less and also those known to be associated a priori. Just a comment.
- Lines 183-184, shorten to: “multicollinearity tests were performed”

Results:

- Lines 201 – 207 – you can just refer to table for characterization of the demographics.
- I had previously suggested combining tables 1 and 2. OK to have separate tables, but I would recommend using bivariate ORs, rather than chi-square tests.
- Lines 226-232 – careful – it’s not 1.93x more likely, but, rather, higher *odds* – this error is repeated.
- Table 2: add 1.0 for the OR for the referent groups. Personally, I don’t think you need p-values if you are giving CIs.

Discussion:

- line 239: you said “1 in 5” before and now saying “2 in 10”
- line 255: type “messaging”

Conclusion:

- lines 297-299: careful about the antenatal screening recommendation - ASCO specifically recommends NOT screening in pregnancy (I disagree with the recommendation and write an opinion piece about it which was published in JGO) – I would take a step back here and suggest that one approach to increasing the provider-pt health education could be to utilize times when reproductive age women are already interfacing with healthcare, like pregnancy.
- Be careful talking about health insurance coverage in Uganda – tie the sentiment to your findings, that “rich” women were more likely to get screened – thus, your recommendation is for screening opportunities to be expanded specifically for poor women – If you are going to talk about national financing for the health sector, this is a huge topic and I would likely rather just hint at it here.
- Seems like your main findings were that rich women, women who learn from providers and women with high knowledge were more likely to screen. But seems like the conclusion only really touches on the first 2 – do you want to somehow also bring in the knowledge variable here?
