# Supplementary material for: Predictors of cervical cancer screening uptake in two districts of Central Uganda
Source: PLoS One. 2020 Dec 3;15(12):e0243281. doi: 10.1371/journal.pone.0243281 (PMC7714132; doi:10.1371/journal.pone.0243281)
Supplement: Attachment — Submitted filename: .Response to Reviewers.docx [file pone.0243281.s007.docx]

**Response letter**

| **Date** | 15 November 2020 |
| --- | --- |
| **To:** | PLOS ONE <plosone@plos.org |
| **From:** | "Alone Isabirye" aloneisab@gmail.com |
| **Subject:** | Response to review comments of our manuscript submitted to PLOS ONE (PONE-D-20-02899R1) |

PONE-D-20-02899R1
Predictors of Cervical cancer screening uptake in two districts of Central Uganda
Alone Isabirye, Martin Kayitale Mbonye, Betty Kwagala
PLOS ONE

Dear Editor,

Thank you for your reply regarding our manuscript "Predictors of Cervical cancer screening uptake in two districts of Central Uganda" (PONE-D-20-02899R1). We are grateful for the reviewers’ comments. We have revised and modified the manuscripts according to the referees’ critiques. As a consequence, we provide a revised manuscript with the reviewers’ suggestions integrated therein:

**Response to editor’s comments**

-Deposit laboratory protocols in [protocols.io](http://www.google.com/url?q=http%3A%2F%2Fprotocols.io&sa=D&sntz=1&usg=AFQjCNFk01OGntovdK1g5gZcchbvuKd9dA); Not applicable

-Uploading our figure files to the Preflight Analysis and Conversion Engine (PACE) digital diagnostic tool; Not applicable

**Response to reviewer 1’s comments**

The overall comment about the revised manuscript is appreciated.

**Abstract:**

- Lines 13-14 were removed.
- Line 16, we added “to improve screening” after “targeted interventions”
- Line 26, as we are shifting to conclusion, we added the word “Overall,” cervical cancer screening low…
- Lines 29 and 30: we removed “the above findings suggest that” and started the sentence with “Efforts to increase uptake…”

**Background:**

- We removed some cancer-specific statistics from paragraph one especially about Uganda and the Cancer Institute and transitioned quicker to the problem of inadequate screening. Additionally, the paragraph was split into two paragraphs.
- Line 57: we wrote-out “Visual inspection with acetic acid” before VIA
- Line 64: second definition of “LMICs” was removed
- Lines 63-67: The section was revised while considering the proposed recommendations by the reviewer.
- Lines 72-79: Phrase comparing various screening methods was removed.

**Methods:**

- Line 181-182: The comment of casting a wide net in variable consideration is appreciated. We included some variables known to be associated a priori like type of residence. However, almost all variables left out in our multivariate model had p-values far higher than 0.2.
- Lines 183-184, the statement was shortened to: “multicollinearity tests were performed”

**Results:**

- Lines 201 – 207 – The table was referred to.
- We are pleased with your decision to accept the two tables. We also humbly propose that chi-square test is used at bi-variate level. This is because data was analyzed at three levels and the selection of statistical approaches at the respective levels was guided by Bloom’s taxonomy of learning objectives.
- Lines 226-232 – 1.93x more likely was corrected to “higher *odds”* – this error was fixed throughout the manuscript.
- Table 2: 1.0 was added for the OR for the referent groups. The authors preferred presenting the results with both p-values and confidence intervals because these statistical measures complement one another.

**Discussion:**

- Line 239: stating “1 in 5” before and stating “2 in 10” later was systematized by mentioning “1 in 5” throughout.
- line 255: We correctly typed “messaging”

**Conclusion:**

- Lines 297-299: The antenatal screening recommendation was fixed as suggested by the reviewer.
- Our recommendation regarding poor women was fixed as proposed by the reviewer.
- We included high knowledge among our main findings.

**Response to reviewer 2’s comments**

- No comments.

We hope that our modifications render our manuscript in its current form suitable for publication in PLOSONE

Yours sincerely,

Isabirye Alone

aloneisab@gmail.com

**On behalf of the authors**
